# A Novel Function of Nonadecanoic Acid in Regulating Glucose Homeostasis

**DOI:** 10.1002/advs.202509534

**Published:** 2026-02-25

**Authors:** Yanting Hou, Yinghua Ma, Qin Liu, Dingling Ma, Yuxin Tong, Lili Xu, Xiaolong Chu, Jingzhou Wang, Maodi Liang, Mengyuan Zhao, Huizi Zhang, Yurui Su, Jianxin Xie, Cuizhe Wang, Jun Zhang

**Affiliations:** ^1^ Medical College of Shihezi University Shihezi China; ^2^ Laboratory of Xinjiang Endemic and Ethic Diseases of Shihezi University Shihezi China

## Abstract

Circulating odd‐chain fatty acids (OCFAs), such as pentadecanoic acid (C15:0) and heptadecanoic acid (C17:0), inversely associate with metabolic syndrome‐related type 2 diabetes mellitus (T2DM), cardiovascular disease, and all‐cause mortality. However, the physiological function of nonadecanoic acid (C19:0) remains unclear. In this study, we identify an inverse association between plasma C19:0 levels and T2DM in the Kazakh population in Xinjiang, China. Investigations using diet‐induced obese (DIO) and *db/db* mouse models revealed that C19:0 has the potential to improve glucose tolerance and enhance insulin sensitivity. Mechanistically, our data demonstrate that C19:0 acts as an endogenous ligand for GPR120, mediating metabolic benefits both in vitro and in vivo. Further analyses indicate that 2‐hydroxyacyl‐CoA lyase (HACL1) directly participates in the biosynthesis of C19:0, with its expression regulated by peroxisome proliferator‐activated receptor α (PPARα). Elevated palmitic acid (PA) levels in obesity suppress PPARα via miR548ab release, thereby impairing the PPARα–HACL1–C19:0 signaling pathway. Collectively, these findings establish a novel association between C19:0 and T2DM and elucidate a distinct mechanism accounting for reduced circulating C19:0 levels in obesity.

## Graphical abstract

This study identifies the odd‐chain fatty acid C19:0 as an endogenous ligand of GPR120 that promotes glucose homeostasis via Gαq signaling. In obesity, elevated palmitic acid suppresses endogenous C19:0 production through a miRNA–PPARα–HACL1 pathway, offering a promising direction for understanding the role of OCFAs in human health.

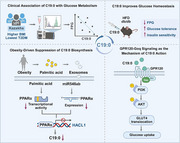

## 1 Introduction

Diabetes is among the nine leading diseases significantly impacting human health and contributing to global mortality rates [1]. Over the past two decades, the global prevalence of diabetes has increased 3.6‐fold, with approximately 90% of cases classified as type 2 diabetes mellitus (T2DM) [2]. Although genetic predisposition partially determines individual susceptibility to T2DM, obesity remains a major driver of its increasing prevalence [3, 4]. Obesity is characterized by increased white adipose tissue (WAT) mass, often accompanied by adipocyte hypertrophy due to excessive triglyceride (TG) accumulation [5]. During obesity, WAT lipolysis increases, leading to excessive release of free fatty acids (FFAs). Higher circulating FFA levels promote ectopic fat deposition, disrupt glucose homeostasis, and induce insulin resistance (IR), ultimately accelerating the progression of T2DM [6, 7]. Hence, FFAs released from adipocytes under obesogenic conditions are considered promising therapeutic targets for regulating impaired glucose homeostasis in T2DM.

FFAs exert diverse physiological functions depending on acyl chain length and degree of saturation [8]. Over the past four decades, elevated circulating even‐chain saturated fatty acid (ECFA) levels have been positively correlated with increased risk of metabolic syndrome, chronic inflammation, T2DM, cardiovascular disease, and other disorders [9, 10]. Palmitic acid (PA) is the most abundant ECFA in dietary sources and in human plasma [11]. It is widely recognized as a principal mediator of lipotoxicity, contributing to metabolic dysfunction through mechanisms such as endoplasmic reticulum stress, mitochondrial impairment, and pro‐inflammatory responses [12]. Conversely, odd‐chain saturated fatty acids (OCFAs)—traditionally considered biomarkers of dairy fat intake—have recently been associated with favorable metabolic outcomes [13]. Accumulating evidence indicates that OCFAs not only originate from dietary sources but can also be synthesized endogenously through α‐oxidation and chain elongation of propionate derived from the gut microbiota [14]. Higher circulating OCFA levels, particularly 15:0 and C17:0, are associated with lower fasting plasma glucose (FPG), improved IR, and reduced T2DM incidence [15].

China has experienced the most rapid increase in T2DM prevalence worldwide [16]. A national cross‐sectional study conducted in 2020 examined the prevalence of diabetes and prediabetes across various regions and ethnic populations in China, identifying multiple contributing factors, including age, obesity, geographic location, and genetic background [17]. Given Xinjiang's unique ethnic composition, we conducted a cross‐sectional analysis among Han, Uyghur, and Kazakh populations. Consistent with previous epidemiological reports [18, 19, 20], Kazakh individuals exhibited the lowest prevalence of diabetes and prediabetes despite having body mass index (BMI) values similar to those of Han and Uyghur individuals. However, the mechanistic basis for this metabolic paradox—low diabetes incidence amid high obesity rates—remains unclear.

In this study, we found that elevated plasma levels of C19:0 may contribute to the low incidence of T2DM in the Kazakh population. We further identified C19:0 as an endogenous ligand for GPR120, capable of exerting hypoglycemic effects via GPR120/Gαq signaling in obese and diabetic mouse models. Additionally, 2‐hydroxyacyl‐CoA lyase (HACL1) was determined to be involved in the in vivo biosynthesis of C19:0, with its expression regulated by peroxisome proliferator‐activated receptor α (PPARα). Elevated PA levels in obesity suppress PPARα activity and expression by promoting miR548ab release, thereby reducing hepatic HACL1 expression. This results in lower circulating C19:0 levels and contributes to IR. Together, these findings reveal a novel association between C19:0 and T2DM and uncover a distinct mechanism underlying obesity‐induced reductions in circulating C19:0 levels.

## 2 Results

### 2.1 T2DM is Associated With Decreased Serum C19:0 Levels

**FIGURE 1:**
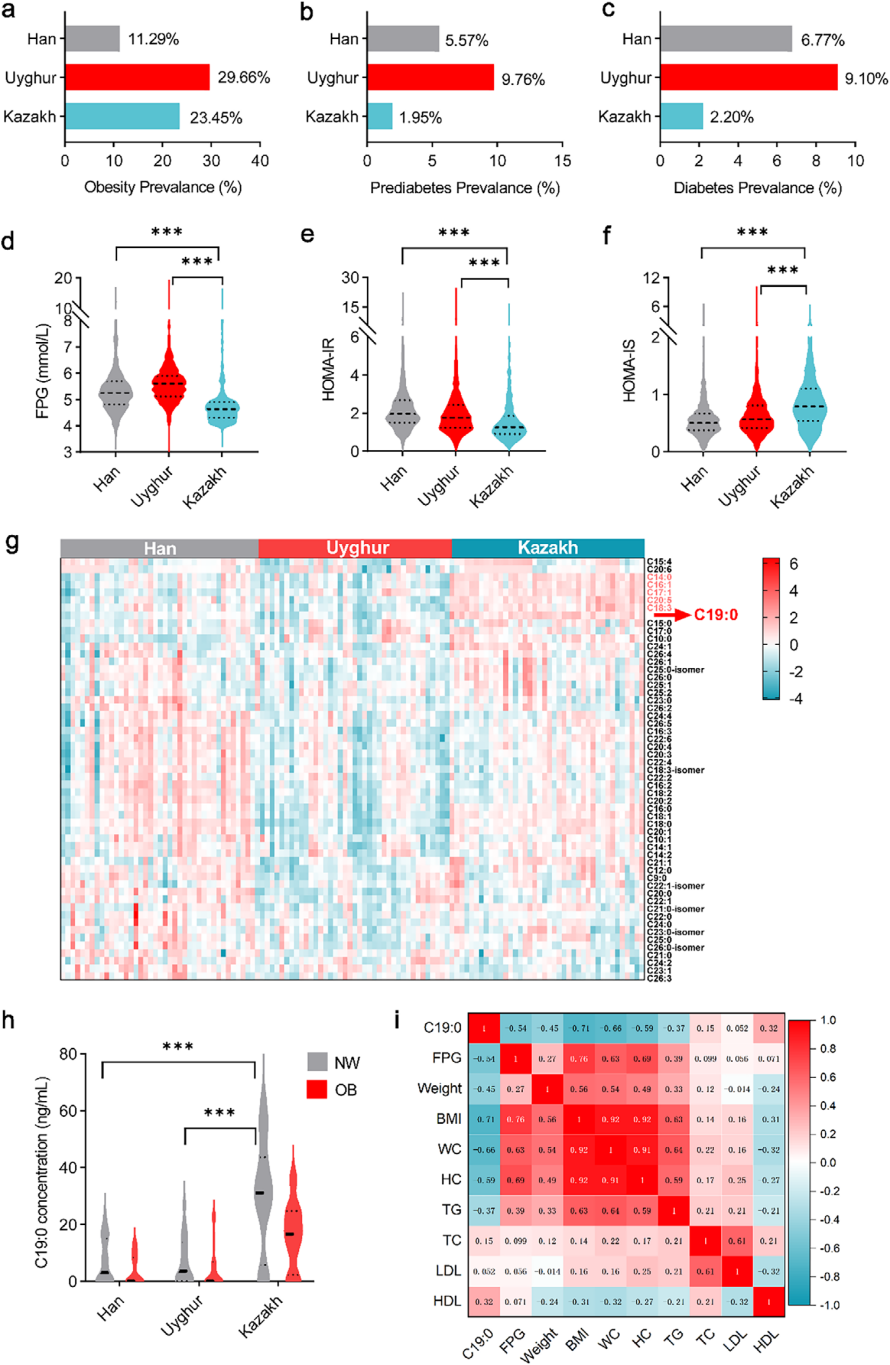
Cross‐sectional analysis of the circulating FFA in associated metabolic phenotypes among three ethnic groups. (a–c) Comparative analysis of the prevalence of obesity (a), prediabetes (b), and diabetes (c) among Han (*n* = 3265), Uyghur (*n* = 11 403), and Kazakh (*n* = 4719) populations. (d–f) Comparative analysis of fasting plasma glucose (FPG) (d), homeostasis model assessment of insulin resistance (HOMA‐IR) (e), and homeostasis model assessment of insulin sensitivity (HOMA‐IS) (f) levels in Han (*n* = 1,340), Uyghur (*n* = 1280), and Kazakh (*n* = 629) populations. (g) Heatmap visualization of free fatty acid (FFA) concentration in Han, Kazakh, and Uyghur groups. (h) Comparison of C19:0 concentration between normal‐weight and obese individuals among Han, Uyghur, and Kazakh ethnic groups. (i) Heatmap of Pearson correlation coefficients between serum C19:0 levels and biochemical indices. Data are presented as violin plots showing the distribution, median, and interquartile range. (d–f, h). ***p < 0.001 compared with the Kazakh group based on the Kruskal–Wallis test followed by Dunn's multiple comparisons test.

We investigated a cohort of 19 387 participants (Han, *n* = 3265; Uyghur, *n* = 11 403; Kazakh, *n* = 4719) to evaluate differences in associated metabolic phenotypes among the three major ethnic groups in Xinjiang, China (Table S1). Cross‐sectional analysis revealed that the Han exhibited the lowest obesity rate (11.29%), whereas the Uyghur and Kazakh populations showed comparable rates (29.66% and 23.45%, respectively; Figure 1a). Notably, the prevalence of prediabetes and diabetes among Kazakhs (1.95% and 2.20%) was markedly lower than that among Han (5.57% and 6.77%) and Uyghurs (9.76% and 9.10%; Figure 1b,c). Serum samples from the three ethnic groups were selected for analysis of key biochemical indicators. Kazakh individuals exhibited significantly lower FPG and homeostatic model assessment of IR (HOMA‐IR) levels, while showing higher homeostatic model assessment of insulin sensitivity (HOMA‐IS) than the Han and Uyghur populations (Figure 1d–f).

Emerging advances in lipid metabolomics have highlighted the critical role of FFA profile heterogeneity in the pathophysiology of T2DM. Notably, while distinct FFA species exert divergent effects on IR and T2DM progression, a systematic approach to deciphering FFA profiles and identifying key metabolic regulatory molecules remains largely unexplored. In this study, we profiled circulating FFA levels in the Kazakh, Han, and Uyghur populations, revealing distinct differences in Kazakh individuals compared with the Han and Uyghur groups (Figure 1g). Importantly, the FFAs that showed significant differences between the Kazakh group and other populations were C14:0, C16:1, C17:1, C20:5, C18:3, and C19:0 (Figure S1). Among these, only C19:0 showed significant differences between individuals with normal weight (NW) and those with obesity (OB) across all three ethnic groups. Moreover, both NW and OB Kazakh individuals had markedly higher serum C19:0 levels than their Han and Uyghur counterparts (Figure 1h). Plasma C19:0 levels exhibited strong inverse correlations with FPG, BMI, and lipid levels (Figure 1i), suggesting a potential protective role of circulating C19:0 in T2DM.

### 2.2 C19:0 is an Endogenous Ligand for GPR120 That Activates Gαq Signaling

Given the observed effects of C19:0 on glucose metabolism and insulin sensitivity, we investigated the molecular mechanisms underlying its action. Many FFAs exert their effects through G protein‐coupled receptors (GPCRs), which play key roles in glucose metabolic regulation. In particular, GPR120 is a receptor for unsaturated long‐chain FFAs, such as DHA [21]. However, it remains uncertain whether OCFAs, given their distinct structural and metabolic properties, function as endogenous ligands. These observed functional similarities prompted an investigation into whether GPR120 may act as a receptor for C19:0.

**FIGURE 2:**
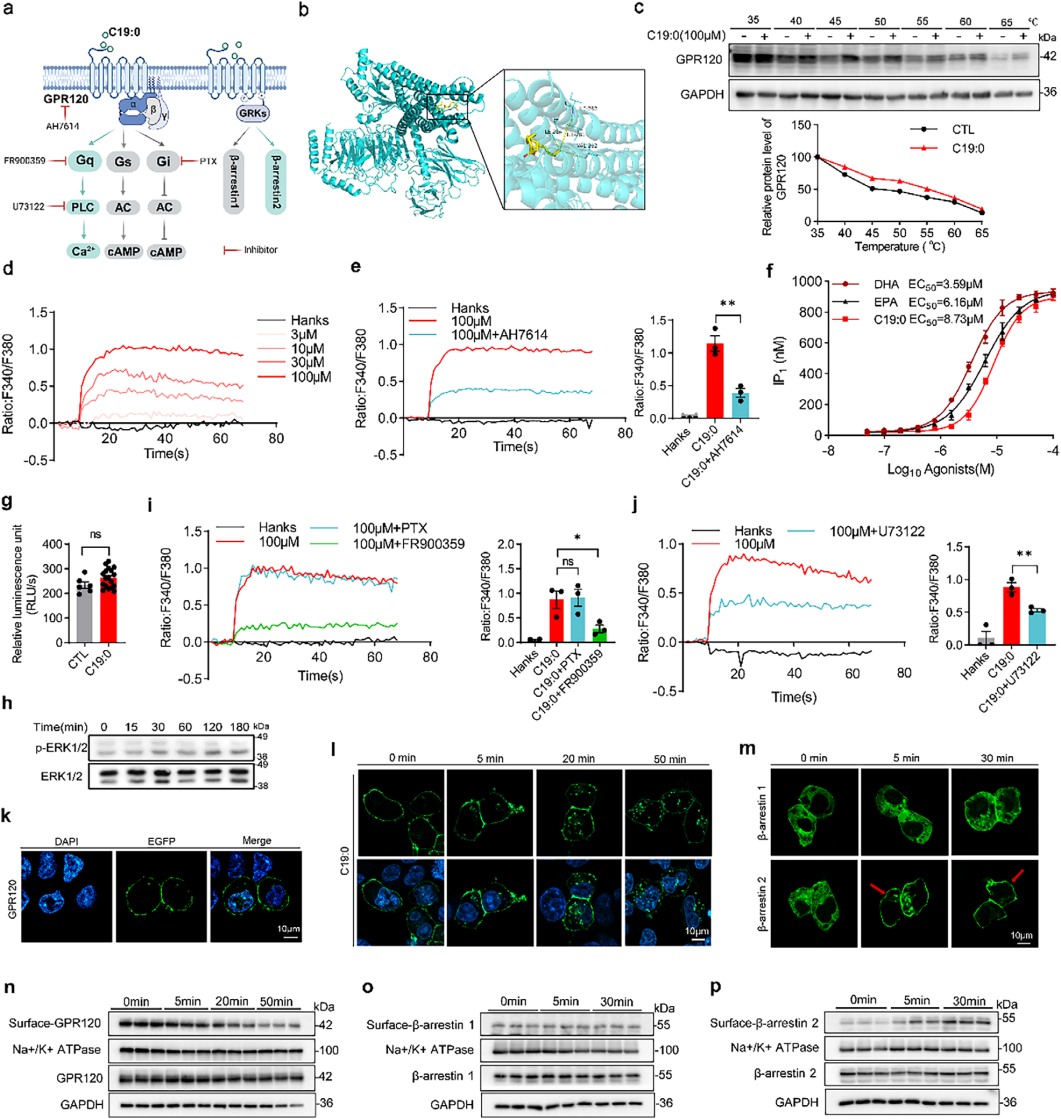
GPR120 is a canonical receptor for C19:0 and activates the Gαq‐dependent signaling pathway. (a) Schematic representation of GPR120 signaling through G protein‐ and β‐arrestin‐mediated pathways, Created in BioRender. (b) Molecular docking analysis of C19:0 binding to GPR120. (c–j) Functional characterization of C19:0 in HEK293T cells overexpressing GPR120. (c) Up: Cellular thermal shift assay (CETSA) analysis of intracellular binding between C19:0 and GPR120. Protein levels were investigated at different temperatures under C19:0 (100 µM) treatment. Down: Quantification for GPR120 protein levels in the CETSA. (d) Intracellular Ca^2^^+^ mobilization in response to C19:0 at different concentrations. (e) Intracellular Ca^2^^+^ mobilization following pretreatment with the GPR120‐specific antagonist AH7614 (10 µM). (f) HEK293T cells were incubated with the indicated concentrations of C19:0, DHA, or EPA for 30 min, and intracellular IP_1_ levels were measured to assess Gαq‐coupled GPR120 activation. (g) HEK293T cells were co‐transfected with p‐CMV‐Flag‐GPR120 and CRE‐Luc reporter plasmids. CRE‐luciferase activity was measured to assess CRE signaling upon C19:0 binding to GPR120. (h) ERK1/2 phosphorylation levels at various time points following treatment with 100 µM C19:0. (i, j) Intracellular Ca^2+^ mobilization after pretreatment of Gαi/o inhibitor PTX (100 ng/mL), Gαq/11 inhibitor FR900359 (1 µM) (i), and PLC inhibitor U73122 (1 µM) (j). (k) Subcellular localization of heterologously expressed GPR120‐EGFP in HEK293T cells in the absence of ligand; Scale bar: 10 µm. (l) Receptor endocytosis in HEK293T cells overexpressing GPR120‐EGFP following treatment with 100 µM C19:0 for different durations; Scale bar: 10 µm. (m) Confocal imaging of HEK293T cells co‐transfected with Flag‐GPR120 and either β‐arrestin1‐EGFP or β‐arrestin2‐EGFP. After 48 h of transfection, cells were stimulated with 100 µM C19:0 for 5 min or 30 min before imaging; Scale bar: 10 µm. (n–p) Cell membrane and total expression levels of GPR120 (n), β‐arrestin 1 (o), and β‐arrestin 2 (p) in HEK293T cells stimulated with 100 µM C19:0 for the indicated times. Data are shown as mean with SEM. *p < 0.05, **p < 0.01 compared with the C19:0 group. Student's *t*‐test was performed in (e, g, j), or one‐way ANOVA test was performed in (i).

A schematic overview of GPR120 signaling through G protein‐ and β‐arrestin‐ mediated pathways is shown in Figure 2a. In silico molecular docking was performed between C19:0 and the human GPR120 protein (PDB ID: 8ID9) using AutoDock Vina 1.1.2. The binding energy of C19:0 to GPR120 was −5.9 kcal/mol, indicating a favorable binding affinity (Figure 2b). Additionally, results from the Cellular Thermal Shift Assay (CETSA) demonstrated that GPR120 exhibits notable thermal stabilization upon exposure to C19:0 (Figure 2c), suggesting a direct binding interaction between C19:0 and GPR120 in living cells.

Treatment with C19:0 elicited a rapid, transient, and dose‐dependent increase in intracellular Ca^2+^ mobilization; this effect was significantly inhibited by pretreatment with the specific GPR120 antagonist AH7614 (Figure 2d,e). To further verify Gαq activation, we quantified inositol monophosphate (IP_1_) accumulation. C19:0 induced a dose‐dependent increase in IP_1_ levels (EC_50_ = 8.73 µM), with an efficacy comparable to that of EPA (EC_50_ = 6.16 µM) but slightly lower than DHA (EC_50_ = 3.59 µM), further supporting its role as a GPR120 agonist (Figure 2f). Since Gαs activates the adenylyl cyclase (AC)–cyclic adenosine monophosphate (cAMP)–protein kinase A (PKA) cascade, leading to phosphorylation of cAMP response element‐binding protein (CREB) [22], we employed a CRE‐luciferase reporter assay to determine whether C19:0 activates Gαs signaling. C19:0 treatment failed to induce CRE activation, suggesting that it fails to trigger Gαs signaling (Figure 2g). Similarly, phosphorylation of ERK1/2 was not detected following C19:0 treatment (Figure 2h). In contrast, the C19:0‐mediated activation of intracellular Ca^2+^ mobilization was blocked by pretreatment with a Gαq inhibitor, FR900359, and the PLCβ inhibitor, U73122, but not by pre‐incubation with pertussis toxin (PTX; Figure 2i,j). These results suggest that C19:0 is an endogenous ligand for GPR120 and activates the Gαq‐dependent signaling pathway.

Ligand‐induced receptor internalization is a key mechanism that directly reflects receptor activation [23]. To visualize GPR120 internalization, we constructed plasmid vectors encoding GPR120 fused to enhanced green fluorescent protein (EGFP) at the C‐terminus. Confocal microscopy revealed that GPR120‐EGFP localized predominantly at the plasma membrane in the absence of ligand (Figure 2k). Upon C19:0 stimulation, GPR120 was rapidly internalized into the cytoplasm (Figure 2l,n). Additionally, using β‐arrestin 1 and β‐arrestin 2 fused to EGFP, we observed significant recruitment of β‐arrestin 2 to the plasma membrane upon C19:0 exposure (Figure 2m,o,p). Collectively, these findings demonstrate that GPR120 is a functional receptor for C19:0, with activation via Gαq signaling and β‐arrestin 2 recruitment.

### 2.3 C19:0 Improves Glucose Homeostasis in Murine Models

**FIGURE 3:**
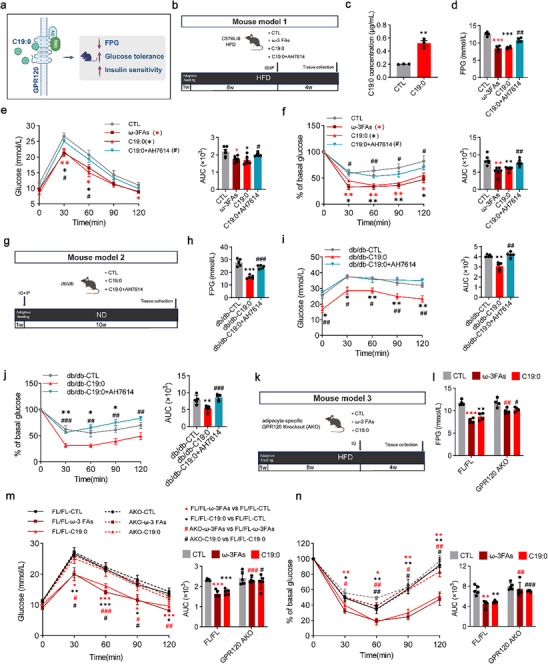
C19:0 is involved in the improvement of glucose homeostasis through GPR120‐mediated signaling. (a) C19:0 acts as an endogenous ligand for GPR120 to exert its hypoglycemic effect, Created in BioRender. (b) Mouse model 1, C19:0 oral administration in HFD mice, mice were treated with vehicle control (CTL), Omega‐3 fatty acids (ω‐3 FAs, 300 mg/kg/day), Nonadecanoic acid (C19:0, 300 mg/kg/day), or C19:0 combined with the GPR120 antagonist AH7614 (5 mg/kg/day). (c) C19:0 levels in serum of mice after oral gavage treatment (*n* = 3). (d) Fasting plasma glucose levels (*n* = 4). (e) Glucose tolerance test (GTT) and its area under the curve (AUC) (*n* = 6). (f) Insulin tolerance tests (ITT) and their AUC (*n* = 6). (g) Mouse model 2, C19:0 oral administration in *db/db* mice. *db/db* mice were treated with vehicle control (CTL), Nonadecanoic acid (C19:0, 300 mg/kg/day), or C19:0 combined with AH7614 (5 mg/kg/day) (*n* = 5). (h) Fasting plasma glucose levels in *db/db* mice. (i, j) GTT and its AUC (i), ITT and its AUC (j). (k) Mouse model 3, C19:0 oral administration in GPR120 AKO mice, mice were treated with vehicle control (CTL), Omega‐3 fatty acids (ω‐3 FAs, 300 mg/kg/day), or Nonadecanoic acid (C19:0, 300 mg/kg/day). (l) Fasting plasma glucose levels in GPR120 AKO mice (*n* = 4). (m, n) GTT and its AUC (m), ITT and its AUC (n) (*n* = 5). Data are shown as mean with SEM. *^,#^ *p* < 0.05, **^,##^ *p* < 0.01, ***^,###^ *p* < 0.001. Student's *t*‐test was performed in (c), one‐way ANOVA test was performed in (d–j), or two‐way ANOVA test was performed in (l–n).

To assess the potential of C19:0 to regulate glucose homeostasis (Figure 3a), high‐fat diet (HFD)‐induced mice and *db/db* mice were supplemented daily with C19:0. Guided by prior studies [15, 24] and our preliminary in vivo toxicity assessments (Figure S2), we selected a 300 mg/kg/day dose for subsequent experiments. After 8 weeks of HFD feeding, the mice were treated with C19:0 (300 mg/kg/day), ω‐3 FAs (positive control, 300 mg/kg/day), or vehicle control for 4 weeks (Figure 3b). No significant differences were observed in body weight (Figure S3a).

Compared to control cohorts, treated animals exhibited significant elevations in serum and WAT C19:0 levels (Figure 3c, Figure S3b), confirming effective systemic distribution. Mice treated with C19:0 exhibited a significant decrease in FPG (Figure 3d), along with improved glucose tolerance and insulin sensitivity (Figure 3e,f). Notably, the glucose‐lowering effects of C19:0 were comparable to those observed with ω‐3FAs treatment, suggesting a similar metabolic benefit. Meanwhile, these improvements were reversed by co‐administration of the GPR120 antagonist AH7614, suggesting that C19:0's beneficial effects on glucose homeostasis were mediated by GPR120 signaling.

To further confirm whether C19:0 has a direct impact on glucose homeostasis, the *db/db* mouse, which carries a G‐to‐T point mutation in intron 18 of the leptin receptor gene, was used as an in vivo model resembling human diabesity [25]. After a 10‐week treatment with C19:0 (300 mg/kg/day; Figure 3g), the *db/db* mice showed no significant differences in body weight (Figure S3c,d). Quantitative analysis revealed significantly elevated C19:0 concentrations in serum samples, demonstrating effective systemic absorption (Figure S3e). The changes in FPG, glucose tolerance, and insulin sensitivity observed in mice were consistent with those exhibited in the HFD mouse model (Figure 3h–j).

The liver, adipose tissue, skeletal muscle, and intestines serve as key metabolic tissues that coordinate systemic glucose homeostasis. However, GPR120 expression is predominantly restricted to adipose tissue and the intestines, with negligible levels in the liver and skeletal muscle [26]. To explore the mechanisms by which C19:0 exerts its effects via GPR120, we generated adipose tissue‐specific GPR120 knockout (GPR120 AKO) mice (Figure 3k). C19:0 administration did not significantly alter food intake (Figure S3f) or body weight (Figure S3g,h) in GPR120AKO mice during the 4‐week intervention. Notably, when challenged with C19:0, GPR120AKO mice exhibited diminished responses in FPG, glucose tolerance, and insulin sensitivity compared to the control group (Figure 3l–n), suggesting adipose GPR120 is essential for mediating C19:0's metabolic benefits.

Despite the significant attenuation of C19:0‐induced metabolic benefits following adipose tissue‐specific GPR120 deletion, the residual improvement in glycemic control suggests the involvement of compensatory mechanisms in other GPR120‐enriched tissues. Notably, GPR120 is abundantly expressed in intestinal enteroendocrine cells [21, 27]. Selective GPR120 activation can promote GLP‐1 secretion, leading to increased circulating insulin levels [28]. To determine whether C19:0 promotes GLP‐1 secretion through intestinal GPR120 activation, thereby facilitating insulin release and contributing to its metabolic benefits even in the absence of adipose tissue GPR120, we examined plasma GLP‐1 levels following C19:0 treatment and assessed the specific role of intestinal GPR120 by using a selective GPR120 antagonist AH7614.

C19:0 administration in HFD‐fed mice elicited a significant elevation in circulating GLP‐1 and higher fasting insulin levels, consistent with the effect of ω‐3FAs (Figure S4a,b). Similar results were observed in *db/db* mice (Figure S4c). Critically, these effects were abolished by AH7614 (Figure S4a,b).

To determine whether C19:0 directly stimulates GLP‐1 secretion, we used the human enteroendocrine NCI‐H716 cells. Exposure to 100 µM C19:0 for 3 h directly induced GLP‐1 secretion in a GPR120‐sensitive manner (Figure S4d), suggesting that intestinal GPR120 has a vital role in C19:0‐induced metabolic benefits. To further confirm this mechanism in vivo, we employed intestinal epithelial‐specific GPR120 knockout (IKO) mice. Under the same treatment conditions, C19:0 failed to elevate plasma GLP‐1 levels in IKO mice, and its effects on glucose tolerance and insulin sensitivity were attenuated compared with flox controls (Figure S4e–i). These findings demonstrate that intestinal GPR120 is essential for C19:0‐induced GLP‐1 secretion and partially accounts for its metabolic benefits.

### 2.4 C19:0 enhances GLUT4 Translocation to the Plasma Membrane via GPR120‐mediated Signaling

**FIGURE 4:**
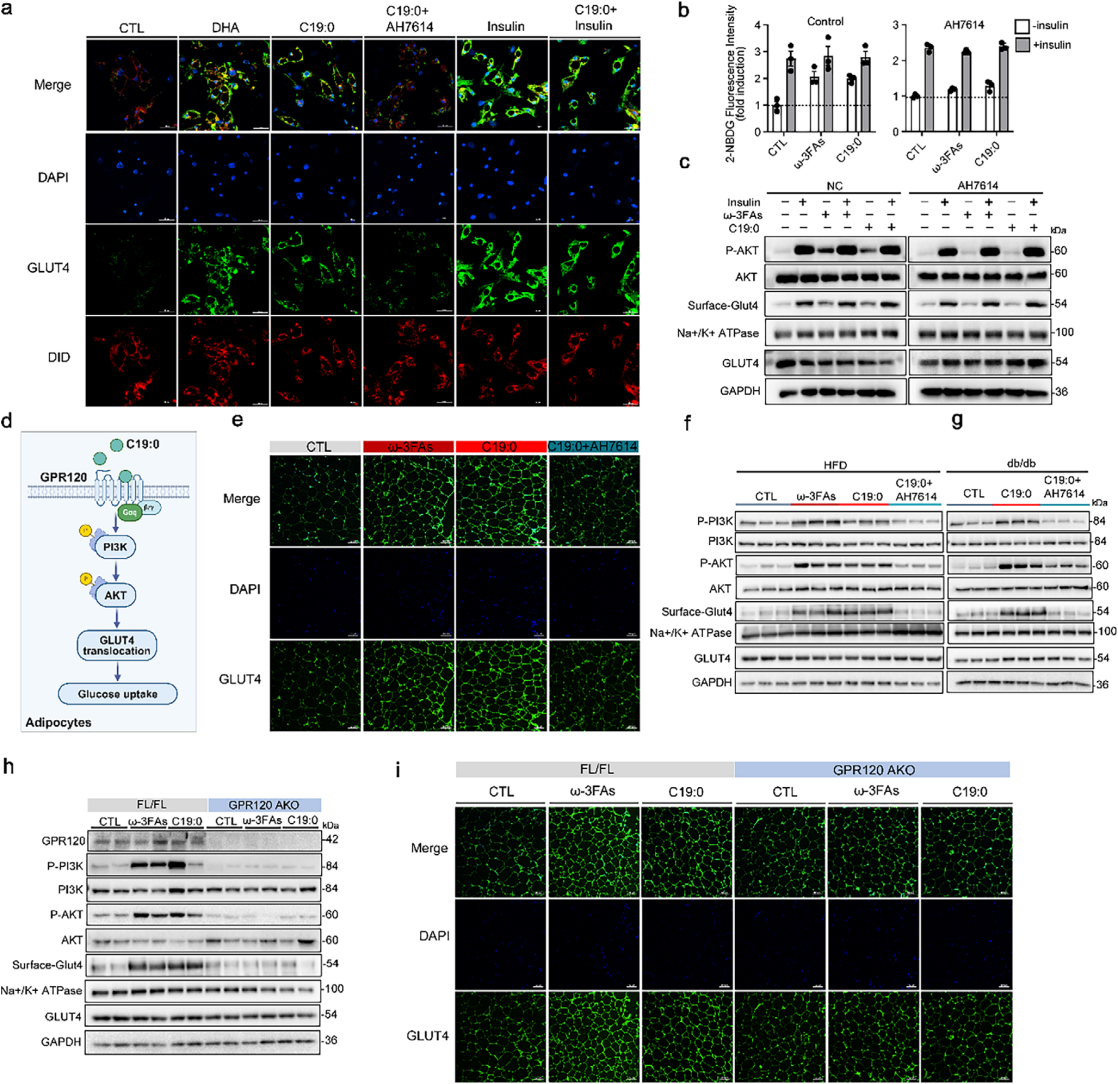
C19:0 enhances GULT4 membrane translocation via GPR120‐dependent AKT signaling cascade. (a) Representative immunostaining of endogenous GLUT4 in primary adipocytes treated with vehicle (CTL), 100 µM docosahexaenoic acid (DHA), 100 µM C19:0, C19:0 + 10 µM AH7614, 100 nM insulin, or insulin + C19:0 for 30 min; Scale bar = 50 µm. (b) Glucose uptake in primary adipocytes pretreated with control or AH7614, followed by treatment with DHA or C19:0, and subsequent stimulation with insulin. (c) Phosphorylated AKT (S473) and membrane GLUT4 expression in primary adipocytes following treatment with 100 µM DHA or 100 µM C19:0. (d) Schematic diagram of GPR120‐mediated GLUT4 translocation in adipocytes, Created in BioRender. (e) Immunofluorescence staining of GLUT4 in WAT of HFD‐induced obese mice; Scale bar = 100 µm. (f) Protein expression levels of phosphorylated PI3K (p‐PI3K), phosphorylated AKT (p‐AKT), and membrane GLUT4 in white adipose tissue (WAT) of HFD‐induced obese mice (*n* = 3). (g) Protein expression levels of p‐PI3K, p‐AKT, and membrane GLUT4 in WAT of *db/db* mice (*n* = 3). (h) Protein expression levels of GPR120, p‐PI3K, p‐AKT, and membrane GLUT4 in WAT of GPR120 AKO mice (*n* = 2). (i) Immunofluorescence staining of GLUT4 in WAT of GPR120 AKO mice; Scale bar = 100 µm.

Glucose transporter type 4 (GLUT4) is a major insulin‐regulated glucose transporter that mediates the removal of circulating glucose [29]. GPR120 has the potential to stimulate GLUT4 translocation to the cell membrane, enhancing glucose uptake in adipocytes [26]. Thus, we assessed the role of C19:0 in regulating GLUT4 translocation. Pretreatment of primary adipocytes with C19:0, docosahexaenoic acid (DHA, a known GPR120 agonist), or insulin (positive control) significantly enhanced GLUT4 translocation to the plasma membrane (Figure 4a) and increased glucose uptake (Figure 4b). This effect was attenuated by pretreatment with AH7614 (Figure 4a,b).

In experiments with exogenous GPR120 expression, C19:0 activated GPR120 and elicited a Gαq‐mediated signal. Previous studies have reported that GPR120/Gαq mediates PI3K–AKT activation in 3T3‐L1 adipocytes [26]. To determine whether C19:0 activates insulin signaling via Gαq in adipocytes with endogenous GPR120 expression, 3T3‐L1 adipocytes were pretreated with the Gαq‐specific inhibitor FR900359 before C19:0 stimulation. C19:0 significantly promoted PI3K and AKT phosphorylation, which was blocked by FR900359 (Figure S4j). Moreover, in primary adipocytes, C19:0 enhanced AKT phosphorylation and promoted GLUT4 membrane translocation, effects that were abolished by AH7614 treatment. (Figure 4c, Figure S4k,l).

C19:0 supplementation enhanced PI3K and AKT phosphorylation and increased GLUT4 translocation in the WAT of HFD and *db/db* mice (Figure 4d–g). Treatment with the GPR120 antagonist AH7614 blocked the beneficial effects of C19:0 (Figure 4e–g). Furthermore, adipocyte‐specific GPR120 knockout abrogated the increase in C19:0‐induced PI3K–AKT phosphorylation and GLUT4 translocation (Figure 4h,i, Figure S4m). These in vitro and in vivo results suggest that C19:0 has the potential to promote glucose uptake via GPR120‐mediated PI3K–AKT–GLUT4 signaling.

### 2.5 Obesity‐Induced Decreases in Circulating C19:0 are Associated With Reduced HACL1 Expression

To investigate the dietary origin of C19:0, we quantified OCFAs in foods commonly consumed by Kazakh individuals, including meat (beef, mutton, and horse meat) and dairy (yogurt lump, ghee, kaymak, mare's milk, and cow's milk) products. Although C19:0 was detectable, its abundance was substantially lower than that of C15:0 and C17:0 (Figure S5a,b). To further evaluate dietary contribution, mice were fed an HFD supplemented with 14% milk fat (HFMF) for four weeks. Among circulating OCFAs, C15:0 levels increased most prominently, followed by a moderate rise in C17:0, while C19:0 remained virtually unchanged (Figure S5c). These results suggest that dietary intake contributes minimally to circulating C19:0, implicating endogenous biosynthesis as the predominant source.

**FIGURE 5:**
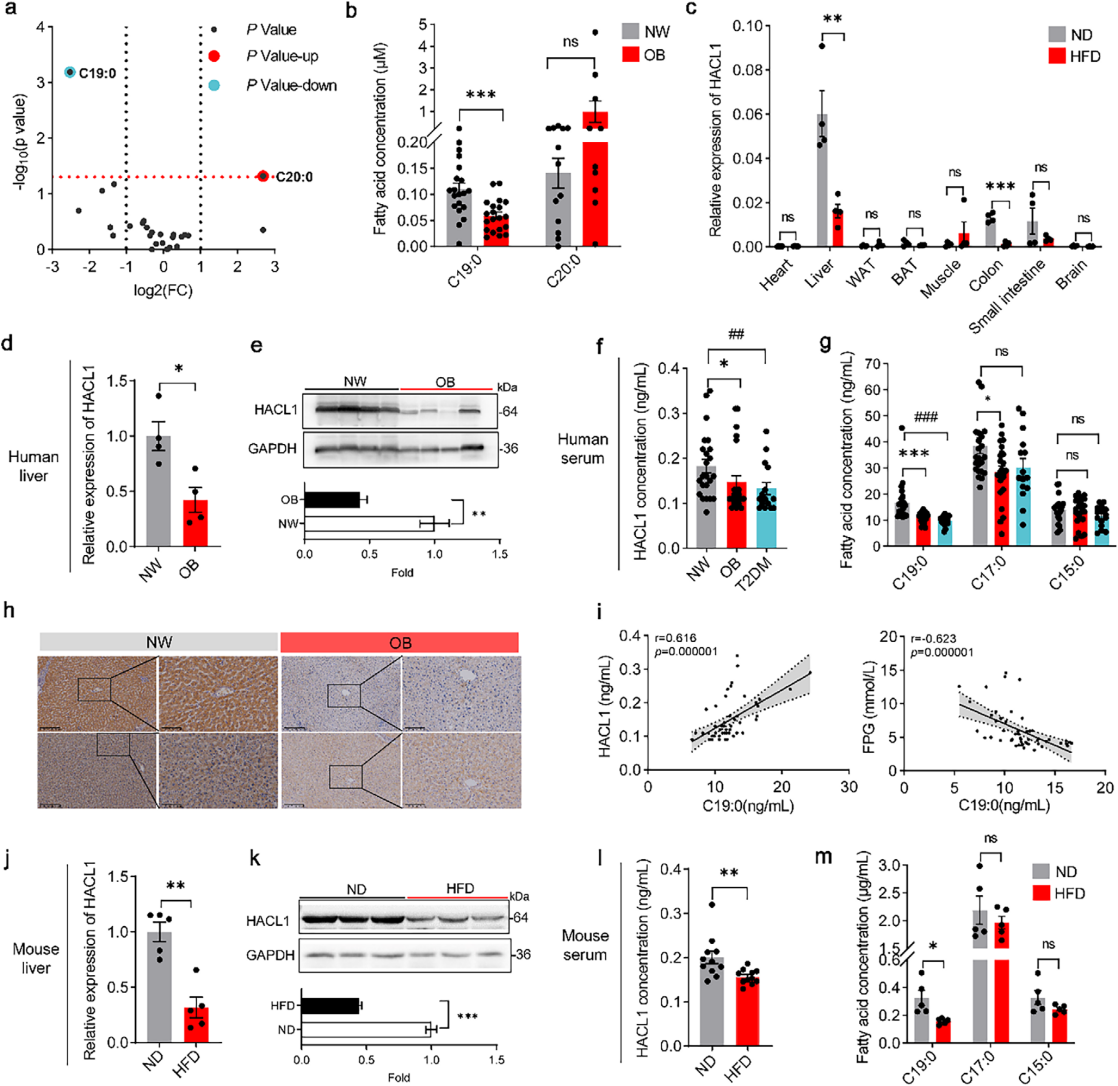
Liver HACL1 is responsible for the obesity‐induced reduction in circulating C19:0. (a) Volcano plot highlighting C20:0 (red) as significantly upregulated and C19:0 (green) as significantly downregulated. (b) Serum levels of C19:0 and C20:0 in normal‐weight (NW) and obese (OB) subjects. (c) qPCR analysis of HACL1 expression in normal diet (ND) and high‐fat diet (HFD) mice (*n* = 4). (d,e) HACL1 mRNA (d) and protein (e) expression levels in the liver of NW and OB subjects (*n* = 4). (f, g) Serum HACL1 levels (f) and concentrations of C19:0, C17:0, and C15:0 (g) in NW (*n* = 23), OB (*n* = 23), and type 2 diabetes mellitus (T2DM, *n* = 15) subjects. (h) Representative immunohistochemical staining of HACL1 in the liver of NW and OB subjects; Scale bar = 200–100 µm. (i) Spearman's correlation analysis of serum C19:0, HACL1 levels, and FPG. Spearman's correlation coefficients (*r*) and *p* values indicate the strength and significance of the associations. (j,k) HACL1 mRNA (j) and protein (k) expression levels in the liver of ND and HFD mice (*n* = 3–5). (l) Serum HACL1 levels in ND and HFD mice (*n* = 11). (m) Serum levels of C19:0, C17:0, and C15:0 in mice (*n* = 5). Data are shown as mean with SEM. *p < 0.05, **^,##^ *p* < 0.01, ***^,###^ *p* < 0.001. Student's *t*‐test was performed in (b–e, j–m), one‐way ANOVA test was performed in (f), or a two‐way ANOVA test was performed in (g).

Plasma FFA profiling revealed a significant decrease in circulating C19:0 and an increase in plasma C20:0 in OB individuals compared with NW controls (Figure 5a,b). HACL1, the key enzyme catalyzing the conversion of ECFAs to OCFAs [30], has been demonstrated by Ampong et al. to mediate the α‐oxidation of C18:0 to generate C17:0. Notably, hepatic HACL1 expression is significantly reduced in HFD‐fed mice [31]. Hence, we hypothesized that HACL1 contributes to the *de novo* biosynthesis of C19:0. To test this hypothesis, we assessed HACL1 expression across tissues and found that it is predominantly expressed in the liver, where its levels were significantly reduced in HFD‐fed mice (Figure 5c). Compared with NW individuals, those with obesity exhibited significantly lower HACL1 expression at the mRNA and protein levels, as evidenced by qRT‐PCR, western blot, ELISA, and immunostaining (Figure 5d–f,h). This aligns with the observation that plasma C19:0 and C17:0 levels were markedly lower in individuals with obesity or T2DM than in NW controls (Figure 5g). As expected, circulating C19:0 levels were positively correlated with hepatic HACL1 expression and inversely associated with FPG (Figure 5i). The same results were observed in HFD‐fed mice (Figure 5j–m, Figure S5d–f).

**FIGURE 6:**
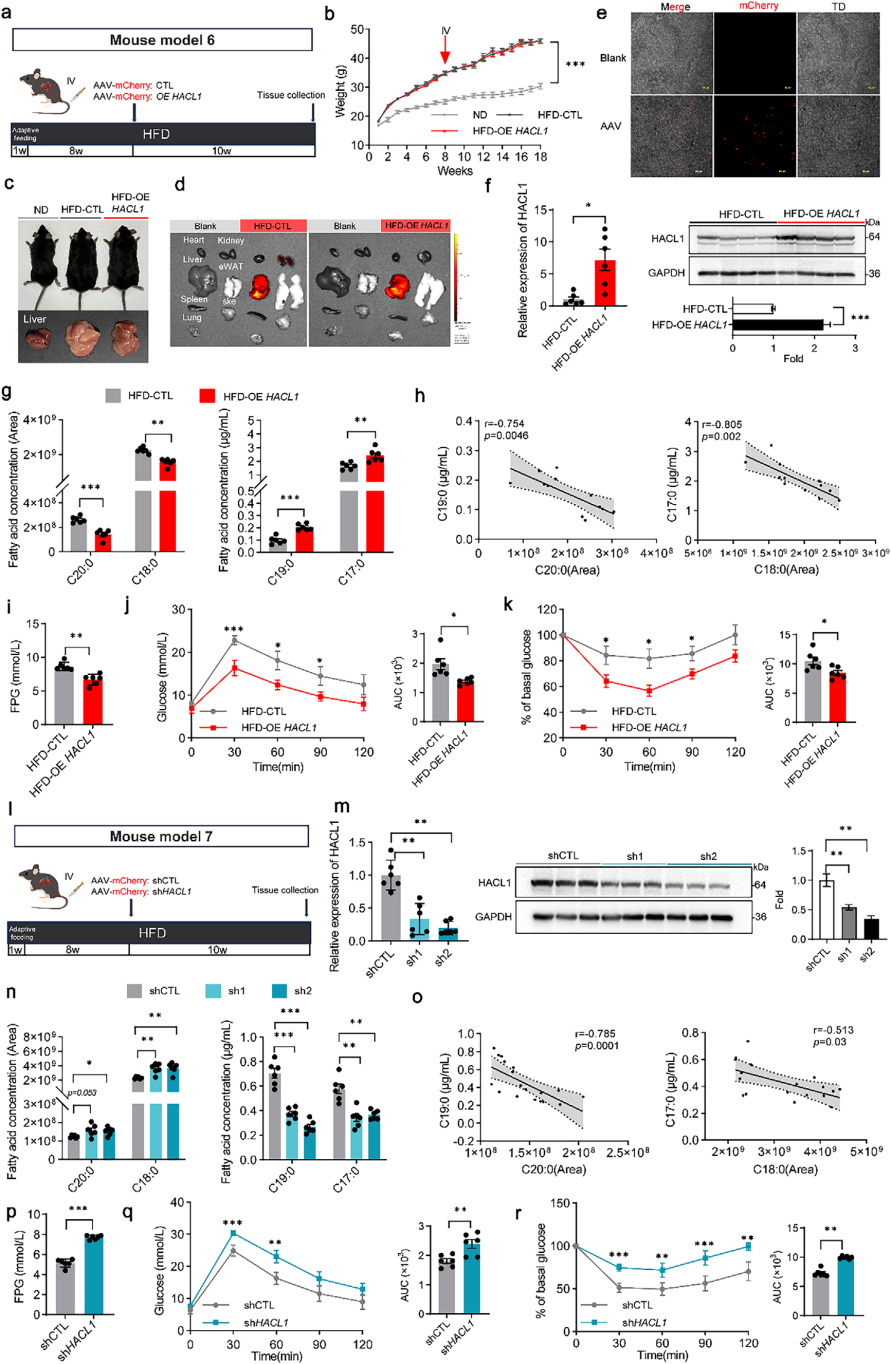
HACL1 is directly involved in the *de novo* synthesis of C19:0. (a) Mouse model 6, Schematic representation of the HACL1 overexpression experiment (*n* = 6). (b) Body weights of mice. (c) Representative images of whole mice and liver morphology. (d) Representative in vivo imaging system (IVIS) images of liver and other tissues after 10 weeks of tail vein injection with mCherry‐labeled adeno‐associated virus (AAV). (e) Representative confocal microscopy images showing AAV localization in the liver; Scale bar = 100 µm. (f) The mRNA (*n* = 6) and protein (*n* = 4) expression levels of HACL1 in liver tissues. (g) Serum levels of C20:0 and C18:0 (quantified by relative abundance), as well as C19:0 and C17:0 (quantified by absolute concentration) in mice. (h) Pearson correlation analysis of serum C19:0 and C20:0, and C17:0 and C18:0. Pearson's correlation coefficients (r) and *p* values indicate the strength and significance of the associations. (i) Fasting plasma glucose levels in HACL1 overexpression mice. (j, k) GTT and its AUC (j), ITT and its AUC (k). (l) Mouse model 7, Schematic representation of the HACL1 knockdown experiment (*n* = 6). (m) The mRNA (*n* = 6) and protein (*n* = 3) expression levels of HACL1 in liver tissues. (n) Serum levels of C20:0 and C18:0 (quantified by relative abundance), as well as C19:0 and C17:0 (quantified by absolute concentration) in mice. (o) Spearman's correlation analysis of serum C19:0 and C20:0, and C17:0 and C18:0. Spearman's correlation coefficients (*r*) and *p* values indicate the strength and significance of the associations. (p) Fasting plasma glucose levels in HACL1 knockdown mice. (q, r) GTT and its AUC (q); ITT and its AUC (r). Data are shown as mean with SEM. *p < 0.05, **p < 0.01, ***p < 0.001. Student's *t*‐test was performed in (b, f, g, i–k, p–r), one‐way ANOVA test was performed in (m), or two‐way ANOVA test was performed in (n).

Next, mCherry‐labeled HACL1 adeno‐associated virus (AAV)‐based overexpression mice were used to confirm the role of HACL1 in *de novo* synthesis of C19:0. Mice fed an HFD for 8 weeks were injected with a mCherry‐labeled AAV expressing HACL1 (Figure 6a). Body weight was unaffected by HACL1 overexpression (Figure 6a–c, Figure S5g). The liver‐targeted overexpression of mCherry‐HACL1 was confirmed (Figure 6d,e, Figure S5h). Consistently, HACL1 expression was successfully upregulated in the liver (Figure 6f). As expected, HACL1 overexpression led to decreased plasma levels of C20:0 and C18:0, and significantly increased levels of C19:0 and C17:0 (Figure 6g,h). Further analysis revealed that plasma FPG, TG, and total cholesterol (TC) levels were significantly downregulated (Figure 6i, Figure S5i), while glucose tolerance test (GTT) and insulin tolerance test (ITT) results were significantly improved (Figure 6j,k).

Subsequently, an AAV‐short‐hairpin RNA (shRNA)‐mediated hepatocyte‐specific HACL1 knockdown mouse model was generated for in vivo experiments (Figure 6l, Figure S5l). HACL1 knockdown led to a significant reduction in HACL1 expression in the livers of mice fed a 12‐week HFD (Figure 6m) and had minimal impact on body weight (Figure S5j,k). HACL1 knockdown led to C20:0 accumulation and reduced circulating C19:0 (Figure 6n,o), along with impaired glucose and insulin tolerance and higher TG levels in serum and the liver (Figure 6p–r, Figure S5m). Collectively, these findings suggest that HACL1 plays a crucial role in the *de novo* synthesis of C19:0.

### 2.6 Palmitic Acid Reduces HACL1 Expression by Inhibiting PPARα Transcriptional Activity

**FIGURE 7:**
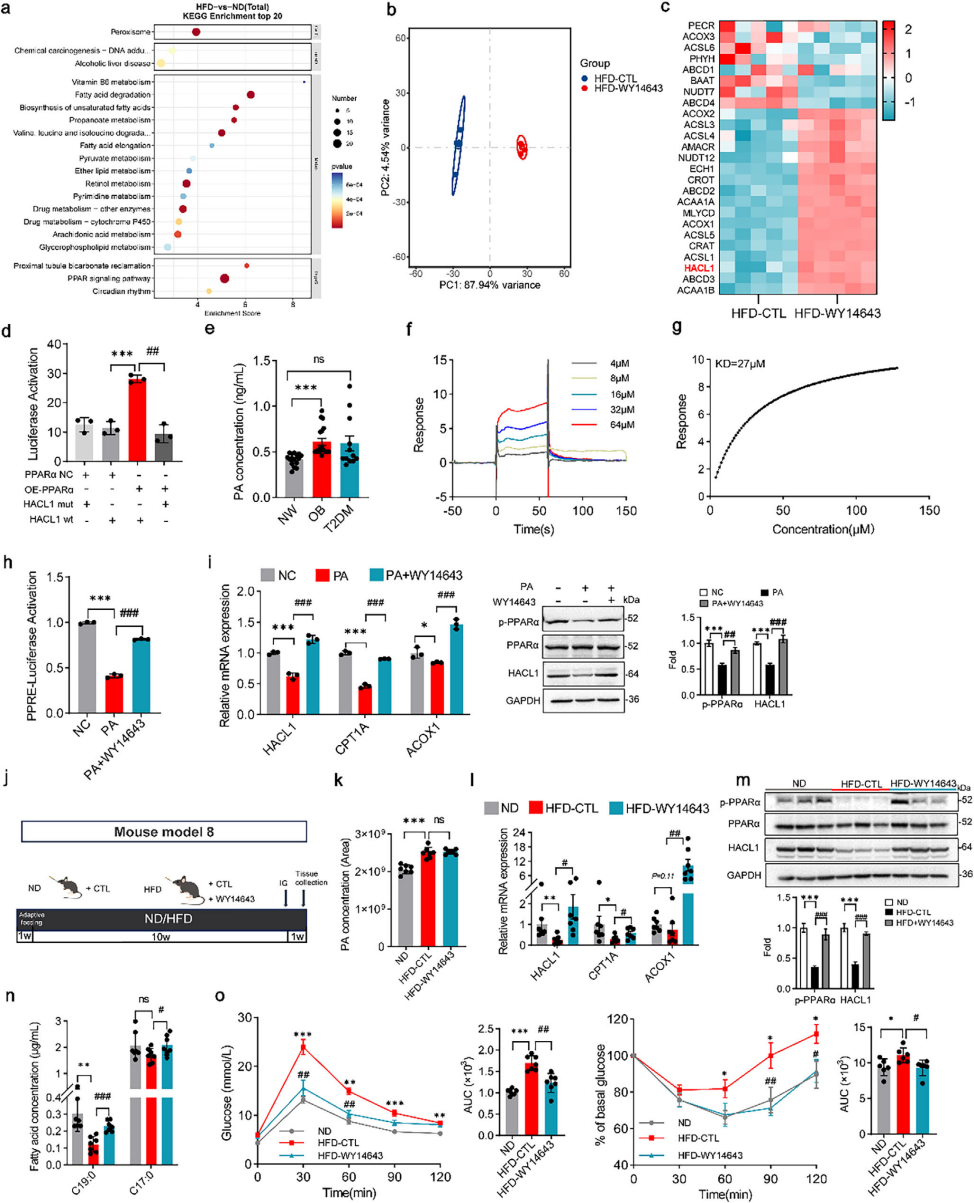
Palmitic acid downregulates HACL1 expression by inhibiting PPARα transcriptional activity. (a) KEGG pathway enrichment analysis of differentially expressed genes (ND vs. HFD). (b) Principal coordinate analysis (PCoA) of RNA‐sequencing data (HFD vs. HFD+WY14643, *n* = 5). (c) Heatmap of differentially expressed genes between HFD and HFD+WY14643‐treated groups (*n* = 5). (d) Luciferase reporter assay in HEK‐293T cells showing PPARα‐mediated activation of HACL1 promoter activity. (e) Serum palmitic acid (PA) levels in human subjects. (f,g) A surface plasmon resonance (SPR) assay showing the affinity of PPARα for PA. The original response curve (f) and corrected response curve (g) are shown. (h) Luciferase reporter assay assessing PA‐induced PPARα transcriptional activity using a PPAR response element (PPRE) reporter in HepG2 cells. (i) mRNA expression of HACL1, CPT1A, and ACOX1 (PPARα target genes) and protein levels of phosphorylated PPARα (p‐PPARα) and HACL1 in HepG2 cells treated with 200 µM PA ± 50 µM WY14643. (j) Mouse model 8, WY14643 treatment experiment (*n* = 7). (k) Serum PA levels in mice measured by UHPLC‐MS (quantified by relative abundance). (l) Hepatic mRNA expression of HACL1, CPT1A, and ACOX1 in obese mice treated with WY14643. (m) Protein expression and quantification of p‐PPARα and HACL1 in liver tissues. (n) Serum C19:0 and C17:0 levels in mice. (o) Left: GTT and its AUC; Right: ITT and its AUC. Data are shown as mean with SEM. *^,#^ *p* < 0.05, **^,##^ *p* < 0.01, ***^,###^ *p* < 0.001. One‐way ANOVA test was performed in (d, e, h, k, o), or two‐way ANOVA test was performed in (i, l–n).

We next explored the molecular mechanisms underlying reduced HACL1 expression in obesity. RNA‐seq of liver tissues from normal diet (ND) and HFD‐fed mice revealed significant alterations in the PPAR signaling pathway, as identified by KEGG pathway analysis (Figure 7a). PPARα is a ligand‐activated transcription factor that regulates genes involved in fatty acid metabolism [32]. RNA‐seq of livers from HFD and HFD + WY14643 (PPARα agonist)‐treated mice showed clear separation between groups in principal component analysis (PCA), with significant upregulation of HACL1 and ACOX1 following WY14643 treatment (Figure 7b,c).

Luciferase reporter assays showed that PPARα overexpression significantly increased HACL1 promoter activity (Figure 7d, Figure S6a). HACL1 expression was modulated by PPARα overexpression or downregulation (Figure S6b–i). These results suggest that PPARα positively regulates HACL1 transcription.

PPARα activity is modulated by various endogenous or naturally occurring biomolecular ligands, including various fatty acids and fatty acid derivatives [33]. Accordingly, primary mouse hepatocytes from HFD mice were treated with a physiologically relevant mixture of FFAs. Exposure to this obesity‐associated FFAs mixture reduced PPARα transcriptional activity (Figure S6j), indicating that fatty acid binding to PPARα does not necessarily lead to transactivation, consistent with previous reports [34].

We next assessed the effects of individual fatty acids on PPARα activity in hepatocytes. PA suppressed PPARα activity, followed by stearic acid, whereas linoleic acid exerted minimal effect, and oleic acid enhanced PPARα activation (Figure S6k). In the context of obesity, enhanced lipolysis elevates circulating levels of PA (Figure 7e), which represents the most abundant FFA. To validate the direct interaction between PA and PPARα, we performed surface plasmon resonance (SPR) assays. PA bound directly to PPARα with an extracellular binding affinity of 27 µM (Figure 7f,g), indicating that PA could serve as a ligand for PPARα. Dual‐luciferase reporter assays confirmed that PA strongly inhibited PPARα transactivation (Figure S6l,m). After treatment with 200 µM PA, CPT1A, ACOX1, HACL1, and p‐PPARα expression was decreased (Figure S6n,o). In contrast, treatment with PPARα agonists increased HACL1 and p‐PPARα levels (Figure S6p–r). Ultimately, administering WY14643 with PA reversed the inhibitory effect of PA on PPARα activity (Figure 7h,i).

Subsequently, to investigate the potential inhibitory effect of PA on PPARα activity in vivo, mice were fed an HFD supplemented with 5% PA (Figure 7j). As anticipated, high‐fat feeding led to significant increases in body weight and liver and adipose tissue weights (Figure S6s–u). With increased serum PA levels in HFD‐fed mice (Figure 7k), liver p‐PPARα, CPT1A, ACOX1, and HACL1 expression decreased (Figure 7l,m). Serum fatty acid profiling further revealed a marked decrease in circulating C19:0 levels, correlating with impaired glucose tolerance and insulin sensitivity (Figure 7n,o, Figure S6v). Additionally, serum and liver TG and TC levels were significantly elevated (Figure S6w). Notably, oral administration of the PPARα agonist WY14643 reversed the inhibitory effect of PA on PPARα activity. This resulted in increased expression of p‐PPARα and HACL1, elevated C19:0 concentration, and improved glucose homeostasis in HFD‐fed mice (Figure 7l–o).

To further establish the causal link between PPARα activation and C19:0 biosynthesis, we performed a liver‐directed rescue experiment using AAV‐mediated gene delivery. Mice received AAV‐control, AAV‐PPARα, or AAV‐PPARα combined with an shRNA targeting HACL1, and serum C19:0 levels were subsequently quantified. As expected, AAV‐PPARα markedly increased circulating C19:0, whereas concomitant knockdown of HACL1 abolished this increase. These findings demonstrate that the PPARα‐driven elevation of C19:0 is dependent on HACL1, supporting a mechanistic PPARα‐HACL1‐C19:0 axis (Figure S6x).

### 2.7 PA‐Induced Exosomal miR548ab Release Leads to Downregulation of PPARα Expression

**FIGURE 8:**
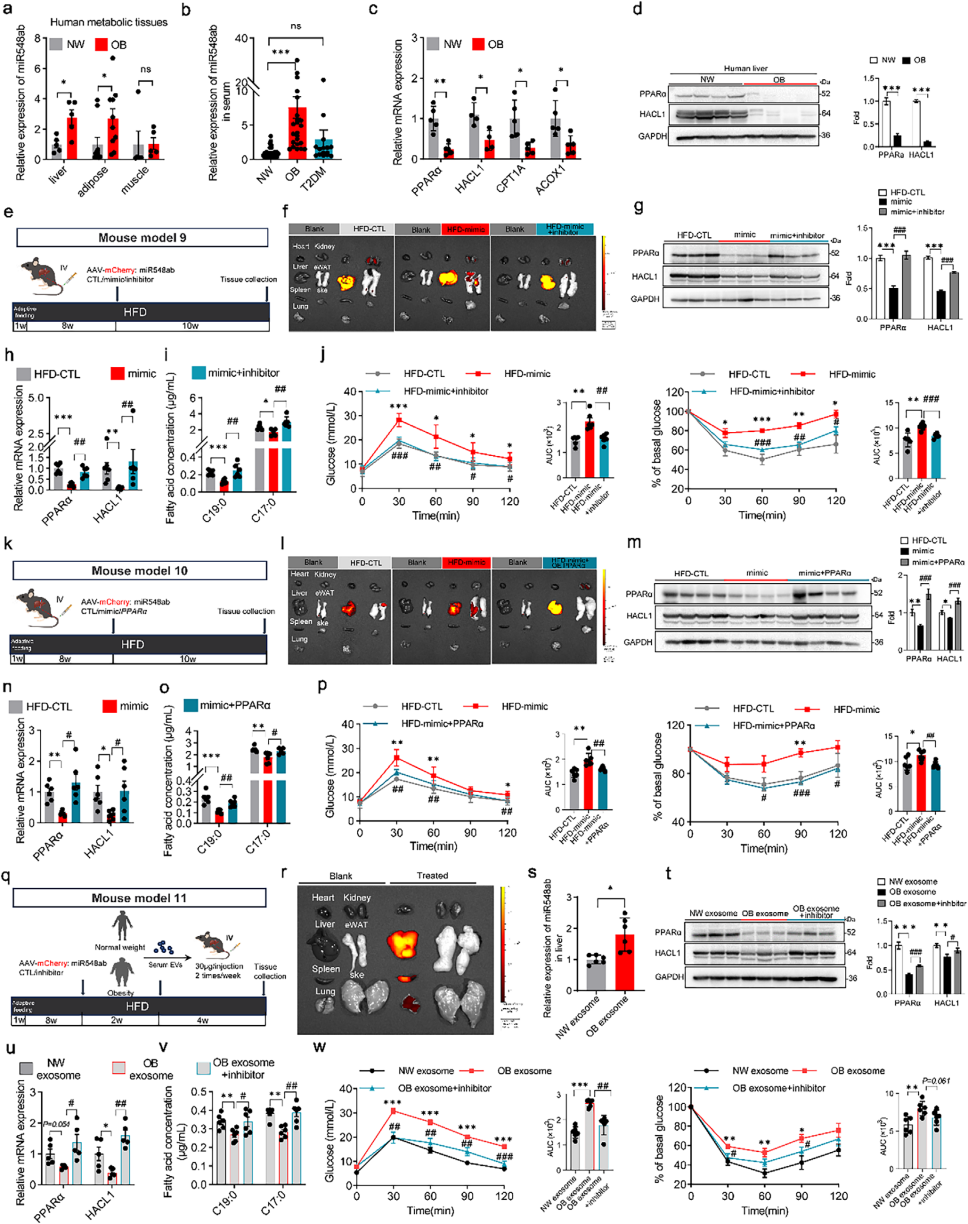
Palmitic acid suppresses PPARα expression through the promotion of the release of exosome‐contained miR548ab. (a) miR548ab expression in liver (*n* = 5), adipose tissue (*n* = 10), and skeletal muscle (*n* = 5) of subjects. (b) Serum miR548ab content in NW (*n* = 25), OB (*n* = 25) and T2DM (*n* = 15) subjects. (c) Hepatic mRNA expression of PPARα, HACL1, CPT1A, and ACOX1 in NW and OB subjects (*n* = 4–5). (d) Protein expression and quantification of PPARα and HACL1 in liver tissue (*n* = 4). (e) Schematic of tail vein injection of adeno‐associated virus (AAV8) expressing mCherry‐tagged miR548ab mimic/inhibitor. (f) Representative IVIS images of liver and other tissues after 10 weeks of tail vein injection with mCherry‐labeled adeno‐associated virus (AAV). (g) Overexpression of miR548ab with simultaneous inhibition in mouse liver: protein expression levels of PPARα and HACL1 (left); quantification (right) (*n* = 3). (h) Hepatic mRNA expression levels of PPARα and HACL1 (*n* = 6). (i) Serum C19:0 and C17:0 levels in mice (*n* = 6). (j) Left: GTT and its AUC; Right: ITT and its AUC (*n* = 6). (k) Schematic of tail vein injection of AAV8 expressing mCherry‐tagged PPARα. (l) Representative IVIS images of liver and other tissues after 10 weeks of tail vein injection with mCherry‐labeled AAV. (m) Overexpression of miR548ab simultaneously up‐regulates PPARα, left: protein expression levels of PPARα and HACL1 (*n* = 4), right: protein quantification. (n) Hepatic mRNA expression levels of PPARα and HACL1 (*n* = 6). (o) Serum C19:0 and C17:0 levels in mice (*n* = 6). (p) Left: GTT and its AUC; Right: ITT and its AUC (*n* = 6). (q) Mouse model 11, serum exosome injection experiment. (r) Representative IVIS imaging of tissue distribution 24 h post tail vein injection of DiD‐labeled serum exosomes. (s) miR548ab content in mouse liver (*n* = 6). (t, u) Hepatic protein (t, *n* = 3) and mRNA (u, *n* = 5) expression of HACL1 and PPARα. (v) Serum C19:0 and C17:0 levels in mice (*n* = 6). (w) Left: GTT and its AUC; right: ITT and its AUC (*n* = 6). Data are shown as mean with SEM. *^, #^ *p* < 0.05, **^,##^ *p* < 0.01, ***^,###^ *p* < 0.001. Student's *t*‐test was performed in (a, c, d, s), one‐way ANOVA test was performed in (b, j, p, w), or two‐way ANOVA test was performed in (g–i, m–o, t–v).

We previously reported that PA promotes the release of exosome‐derived miR548ab from adipose tissue [35], and bioinformatic analysis identified PPARα as a predicted miR548ab target. These findings prompted the hypothesis that elevated PA levels in obesity facilitate the release of miR548ab, thereby suppressing hepatic PPARα expression. Consistent with this, miR548ab levels were significantly increased in the liver, adipose tissue, and serum of obese subjects (Figure 8a,b), whereas PPARα and HACL1 expression were decreased in the liver (Figure 8c,d). Direct binding of miR548ab to the 3′‐untransated region (UTR) of PPARα was confirmed by luciferase reporter assays (Figure S7a,b).

RNA immunoprecipitation (RIP) assays using an anti‐Ago2 antibody confirmed the co‐enrichment of miR548ab and PPARα mRNA in the RNA‐induced silencing complex (RISC), further verifying their direct association in a physiological context (Figure S7c). Furthermore, biotin‐labeled miRNA pull‐down assays provided additional direct evidence of physical binding. The results showed a significantly higher enrichment of PPARα mRNA in complexes pulled down with biotin‐labeled miR548ab compared to those pulled down with biotin‐labeled control (Figure S7d). Significantly, overexpression of miR548ab reduced PPARα levels, whereas miR548ab inhibition increased its expression (Figure S7e,f). Rescue experiments showed that miR548ab‐mediated HACL1 suppression could be reversed by PPARα overexpression (Figure S7g,h). Conversely, inhibition of miR548ab failed to upregulate HACL1 expression when PPARα was concurrently downregulated (Figure S7i,j), suggesting that PPARα mediates the regulatory effects of miR548ab on HACL1.

To evaluate the role of miR548ab in vivo, we delivered mCherry‐tagged AAV encoding miR548ab to the liver via intracerebroventricular injection (Figure 8e). Fluorescence imaging and immunoblotting confirmed elevated hepatic expression of miR548ab in AAV‐treated mice (Figure 8f, Figure S8a), with no significant change in body weight gain compared to HFD‐fed controls (Figure S8b,c). Overexpression of miR548ab led to reduced hepatic PPARα and HACL1 expression, increased hepatic lipid accumulation (Figure 8g,h, Figure S8d,e), decreased serum C19:0 levels, and impaired glucose tolerance and insulin sensitivity (Figure 8i,j). These effects were reversed by co‐administration of a miR548ab inhibitor, which restored OCFA levels and improved metabolic parameters (Figure 8i,j).

To further assess whether miR548ab regulates HACL1 expression via PPARα, we overexpressed miR548ab and PPARα in vivo. The fluorescence imaging and western blot results showed that miR548ab and PPARα, respectively, were successfully enriched in the liver (Figure 8k,l, Figure S8f). Histological analysis showed that miR548ab overexpression promoted hepatic steatosis, whereas co‐overexpression of PPARα ameliorated lipid accumulation (Figure S8g–j). qRT‐PCR and immunoblotting further demonstrated that PPARα overexpression rescued miR548ab‐induced reductions in PPARα and HACL1 (Figure 8m,n). This was accompanied by restored plasma C19:0 levels and improved glucose homeostasis (Figure 8o,p). Taken together, these findings suggest that increased plasma PA exerts its inhibitory effect on *de novo* C19:0 synthesis via the miR548ab‐PPARα‐HACL1 pathway.

To evaluate the role of exosomal miR548ab in the inhibition of hepatic HACL1 expression by targeting PPARα. We isolated exosomes from the serum of NW and OB subjects by ultracentrifugation. Electron microscopy revealed that the isolated serum exosomes were round vesicles approximately 100 nm in diameter (Figure S9a), aligning with the typical exosome size range of 30–150 nm [36]. The quantity of isolated serum exosomes obtained from OB subjects was greater than that observed in NW subjects. Western blot revealed the expression of exosomal markers CD81, CD9, and TSG101 in the isolated serum exosomes (Figure S9b). Further analysis indicated that miR548ab content in the serum exosomes of OB individuals was higher than in NW subjects (Figure S9c). HepG2 cells co‐incubated with serum exosomes from OB subjects exhibited decreased PPARα and HACL1 expression relative to cells treated with NW exosomes (Figure S9d–f), an effect abolished by miR548ab inhibition (Figure S9g,h).

In vivo experiments with HFD‐fed mice treated with isolated serum exosomes from NW or OB subjects were conducted to assess the role of exosomal miR548ab in regulating PPARα and HACL1 expression in the liver. Exogenous exosomes were detected in the liver, resulting in significantly increased miR548ab levels in the liver of OB exosomes‐treated mice (Figure 8q–s). The expression levels of PPARα and HACL1 in the liver of the OB exosome group were significantly lower than those of the NW exosome group (Figure 8t,u). Moreover, treatment with exogenous exosomes isolated from OB subjects resulted in a significant decrease in plasma C19:0 levels (Figure 8v), while serum and liver TG and TC levels were increased, indicating impaired glucose tolerance and insulin sensitivity (Figure 8w, Figure S9j). These effects were significantly reversed by intracerebroventricular injection of miR548ab inhibitor (Figure 8t–w, Figure S9j).

## 3 Discussion

Our epidemiological investigation revealed that, despite a high prevalence of obesity, Kazakh individuals exhibit lower rates of diabetes and prediabetes compared with Han and Uyghur populations. FFAs serve as important sources of energetic substrates and constitute key components of cell membranes [37]. However, FFAs, especially ECFAs such as C16:0 and C18:0, are well‐established risk factors for metabolic syndrome‐related obesity, T2DM, and cardiovascular diseases [38, 39]. On the contrary, a growing body of research provides evidence that plasma levels of OCFAs C15:0 and C17:0 are inversely associated with T2DM, cardiovascular disease, and non‐alcoholic fatty liver disease [39, 40]. In the present study, plasma FFA profiling among Kazakh, Han, and Uyghur individuals revealed distinct ethnic‐specific patterns. Differentially abundant FFAs included C14:0, C16:1, C17:1, C20:5, C18:3, and C19:0. Circulating C19:0 levels were markedly higher in NW and OB Kazakh individuals compared with their Han and Uyghur counterparts, and plasma C19:0 showed strong inverse correlations with FPG, BMI, and lipid levels. To our knowledge, no study has examined the association between circulating C19:0 and obesity‐related metabolic traits.

Beyond their roles as an energy source and a membrane component, FFAs have also been identified over the last 20 years as natural ligands for GPCRs on cell surfaces. These interactions activate signaling pathways that help control metabolic balance and numerous cellular functions. GPR120 (FFAR4) has been identified as a cognate receptor for long‐chain saturated fatty acids (C14–18) and unsaturated fatty acids (C16–22) [21, 41]. However, recent studies on GPR120 have focused on saturated and unsaturated ECFAs, particularly ALA, DHA, and EPA [26]. Whether OCFAs activate GPR120 remains unknown. Our data provide compelling evidence that C19:0 acts as an endogenous ligand for GPR120. Functional characterization demonstrated that C19:0 specifically activates GPR120, eliciting Gαq‐dependent Ca^2+^ mobilization in an AH7614‐sensitive manner, consistent with previous studies [21, 42]. Similar to other OCFAs, the circulating levels of C19:0 detected in human plasma were relatively low; however, low systemic concentrations do not necessarily imply limited biological activity, as localized or receptor‐mediated amplification cascades can convert transient or low‐dose lipid signals into measurable physiological effects, as previously reported for oleoylethanolamide (OEA), palmitoylethanolamide (PEA), and other endogenous lipids such as PAHSAs [28, 43]. Notably, in our IP_1_ accumulation assays, measurable GPR120 activation was observed at concentrations as low as 0.1 µM C19:0, demonstrating that even low‐level C19:0 exposure is sufficient to trigger receptor signaling, albeit with a limited magnitude at these concentrations. Furthermore, in vivo studies using diabetic and obese mouse models demonstrated that C19:0 administration significantly improved glucose tolerance and insulin sensitivity and ameliorated hyperlipidemia. Future investigations employing long‐term administration at physiological concentrations are necessary to more accurately assess the metabolic actions of C19:0. Collectively, these findings identify C19:0 as a previously unrecognized endogenous GPR120 agonist with potent metabolic benefits, highlighting its potential as a pharmacological modulator for improving glucose homeostasis.

Accumulating evidence suggests a pivotal role for GPR120 in improving glucose homeostasis [44]. GPR120 is predominantly expressed in the adipose tissue, intestines, lungs, and pro‐inflammatory macrophages [26]. The high expression of GPR120 in enteroendocrine cells reportedly promotes GLP‐1 secretion and is responsible for the acute glucose regulation of GPR120 agonists [21, 45]. GPR120 agonists directly stimulate insulin secretion via the PLC/Ca^2+^ signaling pathway [28, 46, 47, 48]. In vivo results from the current study suggest that C19:0 may improve glucose homeostasis by enhancing circulating GLP‐1 and insulin concentrations via GPR120‐mediated signaling. Bishop et al. provide evidence that C15:0 can upregulate insulin‐induced AKT phosphorylation [24]. C15:0 is involved in several mechanisms, including the activation of AMPK and PPAR‐α/δ, as well as the inhibition of mTOR, JAK–STAT, and HDAC‐6 pathways. These actions contribute to its diverse biological activities, such as anti‐inflammatory, antifibrotic, and anticancer effects [49, 50]. Holman et al. have reported an inverse association with the development of multiple sclerosis, suggesting that they increase membrane fluidity [51]. Therefore, OCFAs likely exert pleiotropic regulatory effects on various cellular processes and physiological functions. More efforts are required to further elucidate the mechanisms underlying other physiological functions mediated by C19:0.

Higher plasma concentrations of C15:0 and C17:0 are associated with a reduced risk of metabolic diseases, prompting interest in whether OCFAs are endogenously or exogenously derived. Accumulating evidence shows that levels of C15:0 in circulating cholesterol esters positively correlate with dairy product intake [52, 53], and both rumen microbial fermentation as well as intestinal microbial *de novo* lipogenesis contribute to the total plasma OCFA pool [54]. Recent research suggests humans can synthesize OCFAs endogenously from gut‐derived propionate [14, 55]. Plasma C17:0, for instance, is produced endogenously by elongating dietary C15:0 via the enzyme elongation of very long‐chain fatty acid 6 (ELOVL6) [15, 56]. Additionally, α‐oxidation—specifically catalyzed by HACL1 and HACL2—is another pathway for endogenous OCFA production [30, 57]. In alignment with this, our in vivo findings highlight HACL1‐mediated α‐oxidation as crucial for synthesizing 19:0. Although HACL1 predominates in the liver and appears essential for regulating circulating C19:0, it is also expressed in the colon, where its expression is reduced under obese conditions. Thus, intestinal HACL1 may exert a supportive or complementary role in maintaining systemic C19:0 balance. This agrees with the observation that HACL1 knockout resulted in a significant reduction in plasma C17:0 levels [58]. HACL1 has also been suggested as a new candidate gene associated with T2DM and may serve as a potential target for clinical diagnosis and therapeutic intervention [59, 60].

In addition to α‐oxidation, OCFAs may originate from dietary intake or microbial propionate metabolism. However, our quantification of C19:0 concentration in common Kazakh foods, OCFA‐rich dairy products, and HFMF mice suggests that diet only minimally contributes to plasma C19:0; thus, endogenous synthesis likely serves as the principal determinant of circulating C19:0 levels. Moreover, our preliminary observations suggest that gut microbiota‐derived propionate may act as a precursor for OCFA synthesis, thereby influencing circulating C19:0 concentrations (data not shown). Future studies employing germ‐free models and stable isotope tracing will be essential to quantitatively delineate the relative contributions of hepatic α‐oxidation and microbiota‐derived pathways to systemic C19:0 homeostasis and to elucidate their broader metabolic implications.

The present data, obtained from human and murine models of obesity, demonstrate a significant reduction in plasma C19:0 levels relative to non‐obese controls. Therefore, we further explored how elevated FFA levels were associated with lower plasma C19:0 levels. Notably, PA—the most abundant saturated fatty acid in human plasma—increases markedly in IR and diabetes and is strongly linked to metabolic and cardiovascular disease development [12]. Therefore, we focused on the role of PA in regulating endogenous C19:0 production. In obesity, hepatic PA may originate from dietary lipid intake, adipose tissue–derived fatty acids, and endogenous de novo lipogenesis(DNL). In our obese mouse model, expression of key lipogenic genes (Acc, Fasn, and Scd1) was reduced, suggesting that enhanced hepatic DNL is unlikely to be the predominant driver of PA accumulation under these conditions. However, gene expression alone does not permit definitive assessment of relative source contributions, and thus the precise contributions of dietary versus endogenous PA production cannot be distinguished in the present study. Given the chronic lipid oversupply in the HFD model, both dietary fat and increased peripheral lipolysis are likely to substantially contribute to hepatic PA levels.

Elevated PA not only suppressed PPARα transcriptional activity but also stimulated exosomal miR548ab release, which directly targets PPARα and reduces its expression [35, 61]. This combined effect diminishes HACL1 transcription and disrupts endogenous C19:0 biosynthesis, providing mechanistic insight into dysfunction of the PPARα–HACL1–C19:0 axis under lipotoxic conditions. Importantly, PA‐mediated suppression of PPARα activity may not result from a simple direct interaction but instead reflect multiple, potentially convergent mechanisms, including reduced availability of bioactive PPARα ligands such as OEA and PEA, disruption of the balance between PPARα signaling and the endocannabinoid system, and mitochondrial dysfunction and inflammation [43]. Consistent with Herman et al. [34], we observed that PA suppresses PPARα transcriptional activity in a dose‐dependent manner, whereas oleic acid enhances activation at low concentrations, and polyunsaturated fatty acids exert biphasic effects, highlighting that PPARα regulation is context‐dependent and shaped by the integrated lipid environment rather than a single lipid species. In the experiments using a physiological mixture of FFAs, PPARα activity was inferred based on downstream gene expression. Thus, direct assessment of PPARα transactivation would further strengthen these findings and is warranted in future studies.

## 4 Conclusion

In summary, our findings highlight the beneficial role of C19:0 in maintaining glucose homeostasis. Further investigation identified GPR120 as a cognate receptor for C19:0, triggering Gαq‐dependent signaling cascades in response to C19:0 challenge. Furthermore, we identified a specific mechanism underlying increased plasma PA levels in individuals with obesity, involving the downregulation of plasma C19:0 via the miR548ab–PPARα–HACL1 axis. The current study provides a valuable foundation for advancing our understanding of the significance of plasma OCFAs in human health.

## 5 Experimental Section

### 5.1 Human Study

Participant data were collected from hospitals above the county level in 11 cities (or counties) across Xinjiang, including Urumqi, Turpan, Yili, Shihezi, Kashgar, Manas, and Xinyuan, between 2017 and 2018. The data were obtained from health checkups of adults aged ≥ 18 years, including Han (*n* = 3265), Uyghur (*n* = 11 403), and Kazakh (*n* = 4719) individuals, yielding a total sample of 19 387. The study was approved by the Ethics Review Committee of the First Affiliated Hospital, Shihezi University School of Medicine (Approval Number: KJ2021‐020‐01). All participants provided written informed consent before participation.

### 5.2 Mouse Models

C57BL/6J and *db/db* mice were obtained from Changzhou Cavens Laboratory Animal Co., Ltd. Adipocyte‐specific GPR120 knockout (GPR120 AKO) and IKO mice were purchased from Cyagen Biosciences Co., Ltd. All mice were housed in a specific pathogen‐free (SPF) environment with ad libitum access to food and water under controlled conditions. Mice were acclimated for 1 week before the study began. Animals were fed a standard diet (MD12062, Medicience, China) or an HFD (MD12033, Medicience, China). Body weight, body length, and food intake were measured weekly throughout the experiment. All animal experiments were approved by the Medical Ethics Committee of the First Affiliated Hospital of Shihezi University School of Medicine (Approval Number: A2021‐024‐01).

### 5.3 Clinical Sample Collection and Measurements

Participants' weight, height, blood pressure, waist circumference, and hip circumference were measured according to standard operating procedures at the respective hospitals. BMI was calculated as weight (kg) divided by height squared (m^2^). Generalized overweight was defined as a BMI of 24.0–27.9 kg/m^2^, and obesity as a BMI ≥ 28.0 kg/m^2^, for both men and women.

After an overnight fast of at least 10 h, venous blood samples were collected into procoagulant tubes. Serum was separated by centrifugation at 4000 rpm for 10 min. A portion of the serum was used for measuring FPG and 2‐h post‐load plasma glucose following a standard 75‐g oral glucose tolerance test. Additional aliquots were used to assess biochemical parameters, and the remaining serum was immediately stored at −80°C for future analysis.

FPG, fasting plasma insulin (FINS), TC, TG, low‐density lipoprotein cholesterol (LDL‐C), and high‐density lipoprotein cholesterol (HDL‐C) levels were measured using an automated biochemical analyzer. Metabolic syndrome and its components were defined according to the International Diabetes Federation criteria.

The HOMA‐IR and insulin sensitivity (HOMA‐IS) were calculated as follows: HOMA‐IR = (FINS × FPG)/22.5, HOMA‐IS = 22.5/(FINS × FPG)(Note: FPG in mmol/L; FINS in µU/mL).

### 5.4 UHPLC‐MS Analysis

For fatty acid analysis, samples were resuspended in 120 µL of dichloromethane (CH_2_Cl_2_)/methanol (MeOH) (1:1, v/v). Lipid separation was performed using an ultra‐performance liquid chromatography (UPLC) system coupled to an Orbitrap Exploris 240 mass spectrometer (Thermo Fisher, CA, USA) equipped with a heated electrospray ionization (HESI) source. A CORTECS C18 (100 × 2.1 mm, 2.7 µm) column (Waters, USA) was used for chromatographic separation. The mobile phase comprised a binary solvent system: phase A (ACN: H_2_O, 60:40) with 10 mM ammonium acetate, and phase B (IPA: ACN, 90:10). Separation was achieved using an 18‐min linear gradient at a flow rate of 250 µL/min as follows: 0 min, 30% B; 2.5 min, 30% B; 8 min, 50% B; 10 min, 98% B; 15 min, 98% B; 15.1 min, 30% B; and 18 min, 30% B. The column chamber and sample tray were maintained at 40°C and 10°C, respectively.

Mass spectrometric data were acquired in negative ion mode over an m/z range of 150–600, with full‐scan resolution set to 70 000. The ion source parameters were as follows: spray voltage, 3000 V; capillary temperature, 320°C; heater temperature, 300°C; sheath gas flow rate, 35 arb; and auxiliary gas flow rate, 10 Arb.

Lipid identification and data analysis were performed using TraceFinder 3.2 (Thermo Fisher, CA) against an in‐house MS database covering C8–C30 fatty acids. Precursor ion identification was based on accurate mass matching with a 10 ppm mass tolerance. Relative quantification was performed using peak areas, with a 0.25 min retention‐time shift allowance for peak alignment. For key OCFAs (C19:0, C17:0, and C15:0), absolute quantification was performed. For even‐chain fatty acids (C20:0, C18:0, and C16:0), relative quantification was used primarily for trend‐comparison analysis.

### 5.5 Receptor Membrane Localization and Endocytosis Analysis

HEK293T cells transiently expressing GPR120‐EGFP were seeded onto glass coverslips 24 h post‐transfection and cultured overnight. Experiments were conducted 36–48 h post‐transfection. Cells were treated with C19:0 at 37°C, washed with PBS, fixed with 4% paraformaldehyde, and stained with DAPI. The finished images were captured using Fluoview software (Olympus, Tokyo, Japan). Excitation was performed at 488 nm, and fluorescence detection was performed using a 505–530 nm bandpass filter.

### 5.6 β‐Arrestin Recruitment Assay

To study the recruitment of receptors for β‐arrestin1/2, HEK293T cells were co‐transfected with GPR120‐Flag and β‐arrestin1/2‐pEGFP‐N1 plasmids at a 3:1 ratio. The cells were observed under confocal microscopy, photographed, and stored.

### 5.7 Determination of Intracellular Calcium Content

Intracellular calcium flux was detected using the fluorescent calcium indicator Fura‐2/AM. Briefly, pCMV‐Flag plasmids inserted with receptor genes were transfected into HEK293T cells. Cell suspensions were collected by adding PBS buffer containing 0.02% EDTA. Wash two times with Hank's buffer and suspend. Incubate with 3 µL Fura‐2‐AM and 1 µL Pluronic F‐127 surfactant for 30 min at 37°C and wash twice with Hank's buffer. Intracellular calcium fluxes were measured by excitation wavelength ratios of 340 nm and 380 nm over 68 s. If necessary, cells were treated with various inhibitors before the start of the experiment.

### 5.8 HTRF‐Based IP_1_ Accumulation Assay

Intracellular IP_1_ accumulation was measured using the HTRF IP‐One Gq Detection Kit (Revvity) following the manufacturer's protocol. Cells were stimulated with serial concentrations (0.049–100  µM) of C19:0, DHA, or EPA in StimB buffer for 30 min at 37 °C. Following incubation, the IP_1_‐d2 reagent and the IP_1_ Tb cryptate antibody were added. After 1 h, FRET signals (excitation at 340 nm; emission at 620 nm and 665 nm) were recorded using a Varioskan Flash microplate reader (Thermo Fisher Scientific). HTRF ratios were converted to nanomolar concentrations of IP_1_ based on the standard curve.

### 5.9 Mouse Model 1: C19:0 Oral Administration in HFD Mice

Male C57BL/6J mice were fed an HFD for 8 weeks and then randomly assigned to four groups (*n* = 6 per group). Mice continued on the HFD and received oral administration of the following agents for an additional 4 weeks: (1) CTL group: vehicle control (DMSO + Tween‐80 + 0.5% CMC‐Na); (2) ω‐3FAs group: ω‐3 fatty acids (300 mg/kg/day); (3) C19:0 group: C19:0 (300 mg/kg/day); (4) C19:0 + AH7614 group: C19:0 (300 mg/kg/day) combined with AH7614 (5 mg/kg/day, intraperitoneal injection).

### 5.10 Mouse Model 2: C19:0 Oral Administration in db/db Mice

Male *db/db* mice (8 weeks old) were randomly divided into three groups (*n* = 5 per group) and treated for 10 weeks with: (1) *db/db*‐CTL group: vehicle control (DMSO + Tween‐80 + 0.5% CMC‐Na); (2) *db/db*‐C19:0 group: C19:0 (300 mg/kg/day); (3) *db/db*‐C19:0 + AH7614 group: C19:0 (300 mg/kg/day) and AH7614 (5 mg/kg/day, intraperitoneal injection).

### 5.11 Mouse Model 3: C19:0 Oral Administration in GPR120 AKO Mice

GPR120 adipocyte‐specific knockout (AKO) and GPR120 FL/FL mice were fed an HFD for 8 weeks and then randomly assigned to the following groups (*n* = 5 per group) for an additional 4 weeks of treatment: (1) FL/FL‐CTL and AKO‐CTL groups: vehicle control (DMSO + Tween‐80 + 0.5% CMC‐Na); (2) FL/FL‐ω‐3FAs and AKO‐ω‐3FAs groups: ω‐3 fatty acids (300 mg/kg/day); (3) FL/FL‐C19:0 and AKO‐C19:0 groups: C19:0 (300 mg/kg/day).

### 5.12 Mouse Model 4: C19:0 Oral Administration in GPR120 IKO Mice

IKO and GPR120 FL/FL mice were fed an HFD for 8 weeks and then randomly assigned to the following groups (*n* = 5 per group) for an additional 4 weeks of treatment: (1) FL/FL‐CTL and IKO‐CTL groups: vehicle control (DMSO + Tween‐80 + 0.5% CMC‐Na); (2) FL/FL‐C19:0 and IKO‐C19:0 groups: C19:0 (300 mg/kg/day).

### 5.13 Mouse Model 5: Construction of Obese Mouse Model

Six‐week‐old mice were divided into two groups (*n* = 10 per group): (1) Control group: fed a standard diet; (2) HFD group: fed an HFD. After 12 weeks, mice were euthanized, and serum and tissues were collected for further analysis.

### 5.14 Mouse Model 6: HACL1 Overexpression Experiment

Mice fed an HFD for 8 weeks were divided into two groups (*n* = 6 per group): (1) AAV‐Control group: intravenous injection of AAV‐Control (1 × 10^1^ ^2^ vg/mL) for 10 weeks; (2) AAV‐HACL1 group: intravenous injection of AAV‐HACL1 (1 × 10^1^ ^2^ vg/mL) for 10 weeks to induce hepatic HACL1 overexpression.

### 5.15 Mouse Model 7: HACL1 Knockdown Experiment

Mice were fed a standard diet for 8 weeks, then divided into three groups (*n* = 6 per group) and switched to a HFD while receiving the following treatments via intravenous injection for 10 weeks: (1) AAV‐shControl group: AAV‐shControl (1 × 10^1^ ^2^ vg/mL), *n* = 6; (2) AAV‐shHACL1‐1 group: AAV‐shHACL1‐1 (1 × 10^1^ ^2^ vg/mL each); (3) AAV‐shHACL1‐2 group: AAV‐shHACL1‐2 (1 × 10^1^ ^2^ vg/mL each).

### 5.16 Mouse Model 8: WY14643 Treatment Experiment

After 10 weeks of feeding, mice were divided into three groups (*n* = 7 per group): (1) ND group: normal diet‐fed mice treated with vehicle (DMSO + PEG300 + Tween‐80 + saline); (2) HFD‐Vehicle group: HFD‐fed mice treated with vehicle; (3) HFD‐WY14643 group: HFD‐fed mice treated with PPARα agonist WY14643 (50 mg/kg/day) for 1 week.

### 5.17 Mouse Model 9: miR548ab Overexpression Experiment

Mice were fed an HFD for 8 weeks. AAV‐Control and AAV‐miR548ab mimic were administered intravenously 2 weeks in advance (*n* = 6 per group): (1) HFD‐CTL group: AAV‐Control (1 × 10^1^ ^2^ vg/mL) for 10 weeks; (2) Mimic group: AAV‐miR548ab mimic (1 × 10^1^ ^2^ vg/mL) for 10 weeks; (3) Mimic + Inhibitor group: AAV‐miR548ab mimic and AAV‐miR548ab inhibitor (1 × 10^1^ ^2^ vg/mL each) for 10 weeks.

### 5.18 Mouse model 10: miR548ab and PPARα Co‐Overexpression Experiment

Mice were fed with HFD for 8 weeks. AAV‐Control and AAV‐miR548ab mimic were administered intravenously 2 weeks in advance (*n* = 6 per group): (1) HFD‐CTL group: AAV‐Control (1 × 10^1^ ^2^ vg/mL) for 10 weeks; (2) Mimic group: AAV‐miR548ab mimic (1 × 10^1^ ^2^ vg/mL) for 10 weeks; (3) Mimic + PPARα group: AAV‐miR548ab mimic and AAV‐PPARα (1 × 10^1^ ^2^ vg/mL each) for 10 weeks.

### 5.19 Mouse model 11: Serum Exosome Experiment

Mice were fed an HFD for 8 weeks. AAV‐miR548ab inhibitor or control (1 × 10^12^vg/mL) was injected intravenously 2 weeks prior. Three groups were treated for 4 weeks (*n* = 6 per group): (1) NW Exosome group: serum exosomes from NW subjects (30 µg, twice/week, i.v.) + AAV‐miR548ab inhibitor control; (2) OB Exosome group: serum exosomes from OB subjects (30 µg, twice/week, i.v.) + AAV‐miR548ab inhibitor control; (3) OB Exosome + Inhibitor group: serum exosomes from OB subjects (30 µg, twice/week, i.v.) + AAV‐miR548ab inhibitor.

### 5.20 Metabolism Test

For GTT, mice were fasted for 12 h. Baseline body weight and blood glucose levels were measured, followed by intraperitoneal injection of glucose (2 g/kg body weight in saline). Blood glucose was measured at 0, 30, 60, 90, and 120 min using a glucometer (Accu‐CHEK Performa, Roche Diagnostics). For ITT, mice were fasted for 6 h. After recording fasting glucose and body weight, insulin (0.5–1 U/kg body weight) was administered intraperitoneally, and glucose levels were monitored at the same time points as in the GTT. Before sacrifice, mice were fasted for 12 h. FPG levels were measured, and serum samples were collected to quantify FINS levels using ELISA kits (Jianglaibio). HOMA‐IR and HOMA‐IS indices were calculated based on FBG and FINS values.

### 5.21 Western Blot

Equal amounts of protein were denatured in loading buffer by boiling at 100°C and separated on 8%–10% SDS‐PAGE gels. Proteins were transferred to PVDF membranes, blocked with 5% bovine serum albumin (BSA) for 2 h at room temperature, and incubated overnight at 4°C with primary antibodies. Membranes were then washed in TBST and incubated with HRP‐conjugated secondary antibodies for 2 h. Signals were detected using enhanced chemiluminescence (ECL) reagents. Antibody information is provided in the Supplementary Materials.

### 5.22 Real‐Time Quantitative PCR

Total RNA was extracted from liver tissues and cultured cells using TRIzol reagent (Life Technologies), and quantified using a NanoDrop 2000C spectrophotometer (Thermo Fisher Scientific, Waltham, MA, USA). Reverse transcription and qPCR were performed using the RevertAid First‐Strand cDNA Synthesis Kit and SYBR Select Master Mix (Thermo Fisher Scientific), respectively. Primer sequences are listed in the Supplementary Materials. Gene expression levels were normalized to GAPDH or β‐actin and presented as fold changes relative to the control group.

Serum miRNA was isolated using a miRcute Serum/Plasma miRNA Isolation Kit, and the miRcute Plus microRNA First‐Strand cDNA Kit (TIANGEN) was used for reverse transcription. The miRcute plus microRNA SYBR Green qPCR Kit (TIANGEN) was used to detect the expression of microRNA, and Cel‐miR‐39 was used as an external reference for serum/exosomes and U6 as an internal reference for cells or tissues.

### 5.23 Tracking of Labeled AAV and Exosomes

Labeled AAV8 and exosomes localization in various organs and tissues of mice was detected by IVIS Spectrum CT in vivo imaging system (PerkinElmer, USA).

### 5.24 HACL1 Measurement

The level of Human and Mouse serum HACL1 was detected using a HACL1 ELISA kit (Human: FP15519, Mouse: FS52919, Shanghai Westang Bio‐Tech Co., Ltd).

### 5.25 Liver Immunohistochemistry Analysis

The liver tissue slices were stained as follows: slice baking, dewaxing, antigen retrieval, endogenous peroxidase blocking, primary antibody incubation, secondary antibody incubation, DAB chromogenic reaction, hematoxylin counterstaining, dehydration, and sealing. The images were captured using a fully automated digital slide‐scanning system (KF‐PRO‐005‐EX, Ningbo).

### 5.26 Histological Analysis

The liver tissue was fixed in 4% paraformaldehyde and subsequently embedded in paraffin or frozen‐sectioned. The mouse liver tissue was embedded in paraffin and stained with hematoxylin and eosin (H&E) to observe hepatocyte steatosis and inflammation. The frozen‐sectioned liver tissue was stained with oil red O to visualize lipid accumulation. Histological features were examined under a microscope, and images were captured using a fully automated digital slide‐scanning system (KF‐PRO‐005‐EX, Ningbo) for analysis.

### 5.27 Immunofluorescence

Primary mouse adipocytes were seeded on glass coverslips and induced to differentiate according to a standard adipocyte differentiation protocol. Paraffin‐embedded adipose tissue sections were prepared for further analysis. After fixation, samples were blocked with 5% BSA at room temperature for 30–60 min. Coverslips and paraffin sections were then incubated overnight at 4°C with a GLUT4 antibody (Proteintech, Wuhan, China). Following PBST washes, samples were incubated with CoraLite488‐conjugated Goat Anti‐Mouse IgG (H+L) (Proteintech, Wuhan, China) at room temperature for 1 h. Subsequently, nuclei were counterstained with 4′,6‐diamidino‐2‐phenylindole (DAPI) for 10 min. Fluorescent signals were captured using a Nikon AXR laser confocal microscope.

### 5.28 Measurement of TG and TC in Liver and Plasma

The hepatic and serum levels of TG and TC in mice were quantified using a TG and TC quantification kit (Jiancheng, Nanjing, China) following the manufacturer's instructions.

### 5.29 RNA‐Sequencing

Total RNA was extracted using the TRIzol reagent (Invitrogen, CA, USA) according to the manufacturer's protocol. RNA purity and quantification were evaluated using the NanoDrop 2000 spectrophotometer (Thermo Scientific, USA). RNA integrity was assessed using the Agilent 2100 Bioanalyzer (Agilent Technologies, Santa Clara, CA, USA). The libraries were constructed using the VAHTS Universal V6 RNA‐seq Library Prep Kit according to the manufacturer's instructions. The libraries were sequenced on an Illumina Novaseq 6000 platform. OE Biotech Co., Ltd, conducted the transcriptome sequencing and analysis.

### 5.30 Surface Plasmon Resonance (SPR) Assay

SPR analysis was performed using a Biacore T200 instrument (GE Healthcare, Chicago, IL, USA) to determine whether PA could directly bind to PPARα. Recombinant human PPARα protein was immobilized on the CM5 chip (GE Healthcare, Chicago, IL, USA) through 1‐ethyl‐3‐(3‐dimethylaminopropyl) carbodiimide/*N*‐hydroxysuccinimide (EDC/NHS)‐mediated crosslinking reaction. The fatty acid to be tested was dissolved in PBS containing 5% DMSO, with concentrations ranging from 0.5 to 64 µM. The flow rate at the injection chip surface was 30 µL/min, with a contact time of 60 s and a dissociation time of 120 s. After the addition of DMSO, a series of eight concentration gradients ranging from 4.5% to 5.8% were prepared to account for the solvent effect on the final results before and after testing. The steady‐state affinity model was utilized for fitting the affinity curve, and the dissociation constant KD was calculated with Biacore T200 evaluation software 3.0.

### 5.31 Cell Culture

HEK293T, HepG2, and LO2 cells were cultured in Dulbecco's Modified Eagle's Medium (DMEM) supplemented with 10% fetal bovine serum (FBS; Gibco, BRL, Grand Island, NY) and 1% penicillin/streptomycin at 37°C in a 5% CO_2_ incubator. WAT was minced into small fragments and digested in DMEM/F12 medium containing 1 mg/mL collagenase II at 37°C for 30 min. The resulting cell suspension was passed through a 100 µm cell strainer and centrifuged at 800 rpm for 5 min. The isolated stromal vascular fraction (SVF) cells were resuspended and cultured in DMEM/F12 medium supplemented with 10% FBS. Once the cells reached 90% confluence, adipogenic differentiation was initiated by replacing the medium with induction medium containing 10% FBS, 1% penicillin/streptomycin (P/S), 0.5 mM isobutylmethylxanthine (IBMX), 1 µM dexamethasone, 1 µM rosiglitazone, and 5 µg/mL insulin. After 2 days of induction, the medium was replaced with maintenance medium (DMEM/F12 supplemented with 10% FBS, 1% P/S, 1 µM rosiglitazone, and 5 µg/mL insulin). This process was repeated until the cells successfully differentiated into mature adipocytes.

### 5.32 Glucose Uptake

Glucose uptake in primary adipocytes was measured using the fluorescent glucose analog 2‐(N‐(7‐nitrobenz‐2‐oxa‐1,3‐diazol‐4‐yl)amino)‐2‐deoxyglucose (2‐NBDG) (MedChemExpress, New Jersey, USA). Cells were serum‐starved for 4 h, followed by incubation in glucose‐free and phenol red‐free DMEM for 2 h. Cells were then pretreated with the indicated drug for 30 min and subsequently stimulated with 100 nM insulin for 10 min. Thereafter, cells were incubated with 100 µM 2‐NBDG for 20 min. Following two washes with PBS, intracellular 2‐NBDG uptake was detected using a fluorescence microplate reader at Ex/Em = 467 nm/542 nm.

### 5.33 Luciferase Reporter Gene Assays: PPARα‐Mediated Transcriptional Activation of HACL1

HEK‐293T cells were co‐transfected with wild‐type (WT) or mutant (MUT) HACL1 luciferase plasmids and PPARαplasmids, or PPARα control and Renilla luciferase plasmids.

### 5.34 Effects of PA on PPARα Activity

HepG2 cells were co‐transfected with a PPARα plasmid, PPAR response element (PPRE) X3‐TK luciferase plasmid, and Renilla luciferase plasmid. Following transfection for 6–8 h, the cells were treated with 200 µM PA, 10 µM GW6471, or 50 µM WY14643 for 24 h.

### 5.35 miR548ab Targeted PPARα

HEK‐293T cells were co‐transfected with WT or MUT PPARα luciferase plasmids, miR548ab mimic or microRNA negative control, and Renilla luciferase plasmids.

The transfection described above was conducted using Lipofectamine 2000 following the manufacturer's instructions. Luciferase activity assays were performed using the Dual‐Luciferase Reporter Assay System (Promega) and normalized to Renilla luciferase activity.

### 5.36 Isolation and Characterization of Plasma Exosomes

Plasma samples were centrifuged at 2000 × *g* for 15 min, and the supernatant was collected. The supernatant was then centrifuged at 4°C and 10 000 × *g* in a high‐speed refrigerated centrifuge for 30 min, and the supernatant was collected again. The supernatant was then filtered using a 0.22‐µm needle filter. The filtered sample was transferred to an ultracentrifuge tube for the Himac ultracentrifuge and centrifuged at 4°C for 90 min. The supernatant was removed, and the precipitate was resuspended in PBS. The sample was then centrifuged again at 4°C for 90 min; the supernatant was discarded, and the exosome precipitate was resuspended in approximately 160 µL PBS. The exosome samples were diluted with PBS solution to an appropriate concentration. The size distribution analysis was performed using a nanoparticle tracking analyzer (ZetaView, Particle Metrix, Germany), and the exosome morphology was detected by transmission electron microscope (TEM, HT7800/HT7700, HITACHI, Japan).

### 5.37 Statistical Analysis

Results are presented as mean with standard error of the mean (SEM). All bar plots were generated using GraphPad Prism 9.0 (GraphPad Software, La Jolla, CA, USA). Statistical analyses were performed using SPSS 20.0 (SPSS Inc., Chicago, USA). For comparisons between two groups, Student's *t*‐test was applied for normally distributed data, while nonparametric tests were used for non‐normally distributed data. One‐way analysis of variance (ANOVA) and two‐way ANOVA were conducted for comparisons among three or more groups, and Spearman's and Pearson correlation analyses were performed to assess correlations. A *p*‐value < 0.05 was considered statistically significant.

## Author Contributions

J.Z. and C.‐Z.W. conceptualized and designed the study. J.Z. and J.‐X.X. provided funding and supervision. Y.‐T.H., Y.‐H.M., Q.L., and D.‐L.M. performed the experiments. Y.‐X.T. and L.‐L.X. assisted with cell experiments. X.‐L.C., J.‐Z.W., and M.‐D.L. contributed to animal experiments. M.‐Y.Z., H.‐Z.Z., and Y.‐R.S. collected human samples. Y.‐T.H. designed the experiments and analyzed the data. Y.‐T.H. and Y.‐H.M. wrote the manuscript. J.Z. and C.‐Z.W. critically revised the manuscript.

## Conflicts of Interest

The authors declare no conflicts of interest.

## Supporting information




**Supporting File**: advs74580‐sup‐0001‐SuppMat.docx.

## Data Availability

The data that support the findings of this study are available in the supplementary material of this article.
