# Supplementary material for: A Novel Function of Nonadecanoic Acid in Regulating Glucose Homeostasis
Source: Adv Sci (Weinh). 2026 Feb 25;13(26):e09534. doi: 10.1002/advs.202509534 (PMC13159108; doi:10.1002/advs.202509534)
Supplement: Supplementary file 1 — Supporting File: advs74580‐sup‐0001‐SuppMat.docx. [file ADVS-13-e09534-s001.docx]

SUPPPORTING INFORMATION


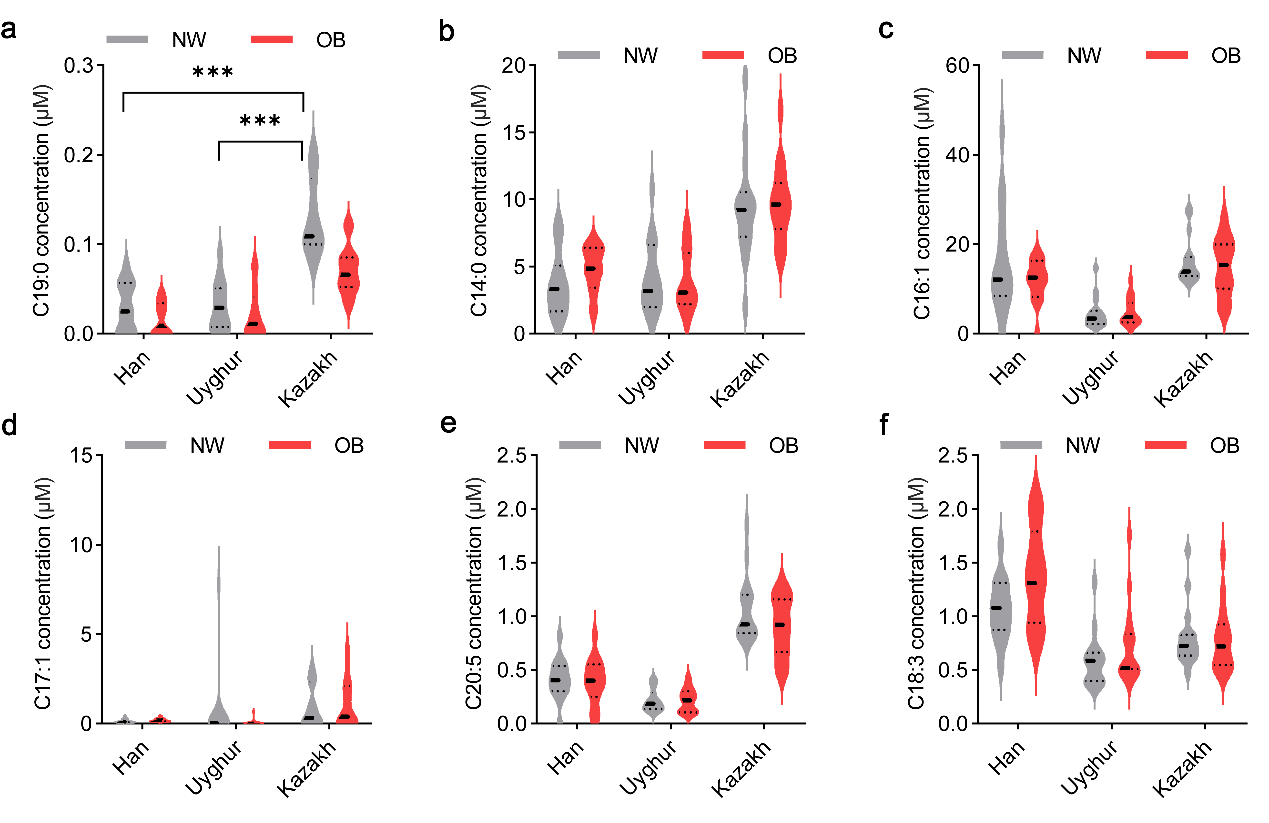


**Figure S1.** Differential fatty acids among the three ethnic groups. (a–f) Comparison of C19:0 (a), C14:0 (b), C16:1 (c), C17:1 (d), C20:5 (e), and C18:3 (f) concentration between normal-weight and obese individuals among Han, Uyghur, and Kazakh ethnic groups.


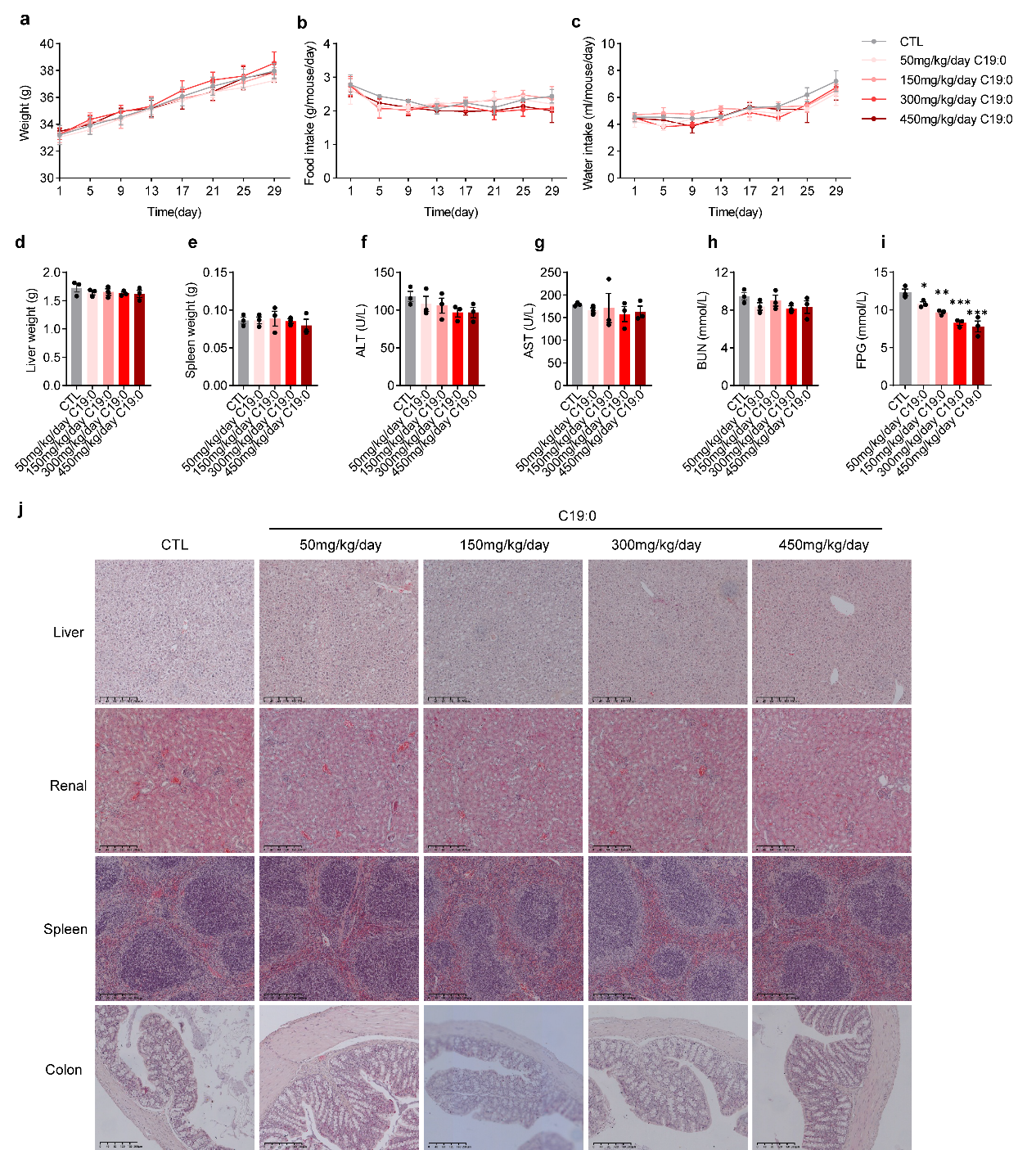


**Figure S2**. Safety assessment of oral C19:0 administration in mice. (a–c) Body weight (a), food intake (b), and water intake (c) of mice during oral administration of C19:0. (d, e) Liver (d) and spleen (e) weights of mice after oral C19:0 treatment. (f, g) Serum alanine aminotransferase (ALT) and aspartate aminotransferase (AST) levels assessing liver function after oral C19:0 treatment. (h) Serum blood urea nitrogen (BUN) levels evaluating renal function after oral C19:0 treatment. (i) Fasting blood glucose levels in mice after oral C19:0 treatment. (j) Representative H&E staining of liver, kidney, spleen, and colon tissues from mice after oral C19:0 treatment. Data are shown as mean with SEM. **p* < 0.05, ***p* < 0.01, ****p* < 0.001, compared with the CTL group, one-way ANOVA test was performed in (i).


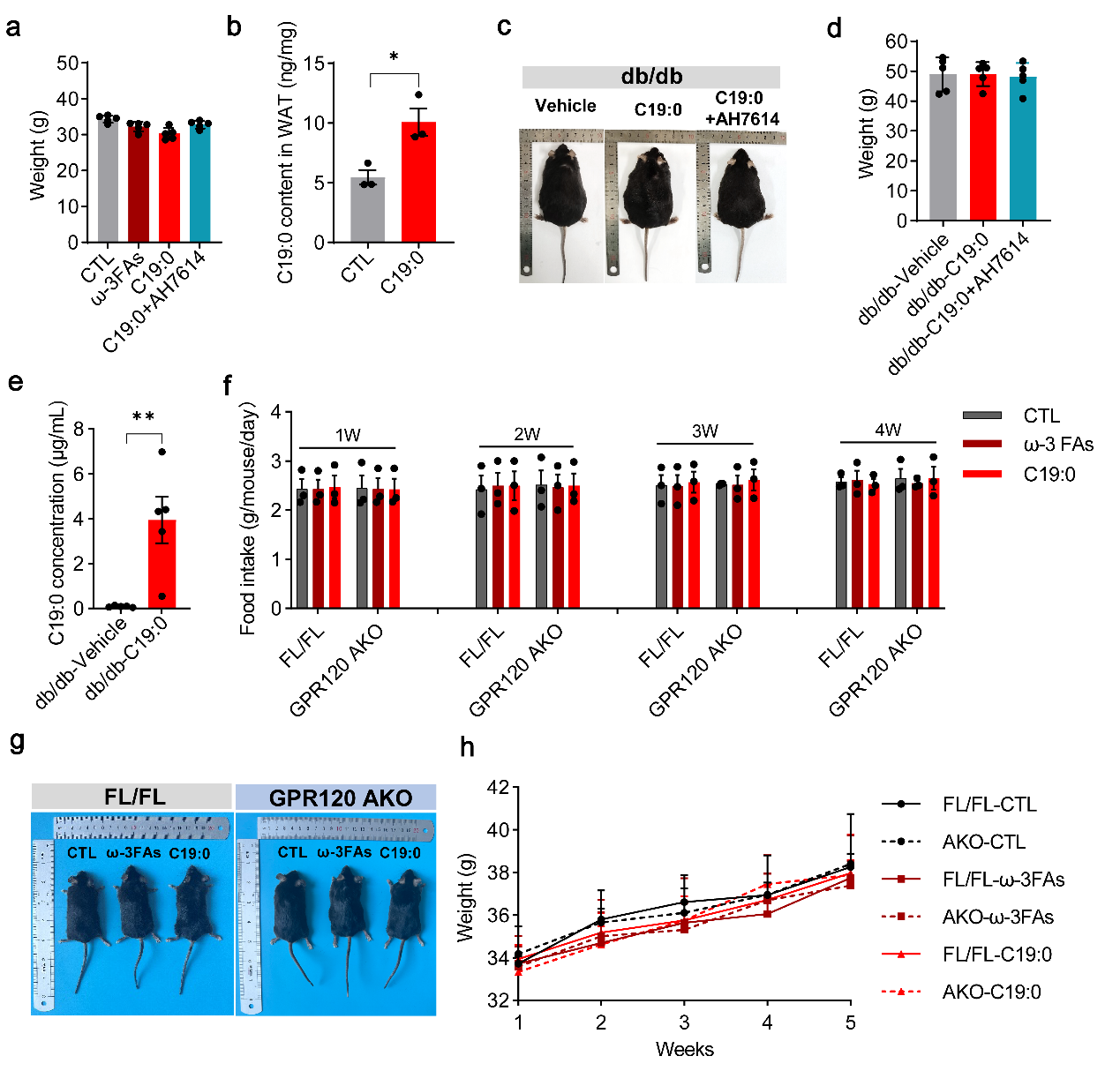


**Figure S3.** Body weight and food intake of C19:0-treated mice. (a) Body weight in HFD mice (*n* = 5). (b) C19:0 levels in white adipose tissue of mice after oral gavage treatment (*n* = 3). (c) Representative whole-body images of *db/db* mice. (d) Body weight in *db/db* mice (*n* = 5). (e) C19:0 levels in serum of *db/db* mice after oral gavage treatment (*n* = 5). (f) Food intake in GPR120 AKO model (*n* = 3). (g) Representative whole-body images of GPR120 AKO. (h) Body weight progression in GPR120 AKO mice (*n* = 5–6). Data are shown as mean with SEM. **p* < 0.05, ***p* < 0.01. Student’s *t*-test was performed in (b, e).


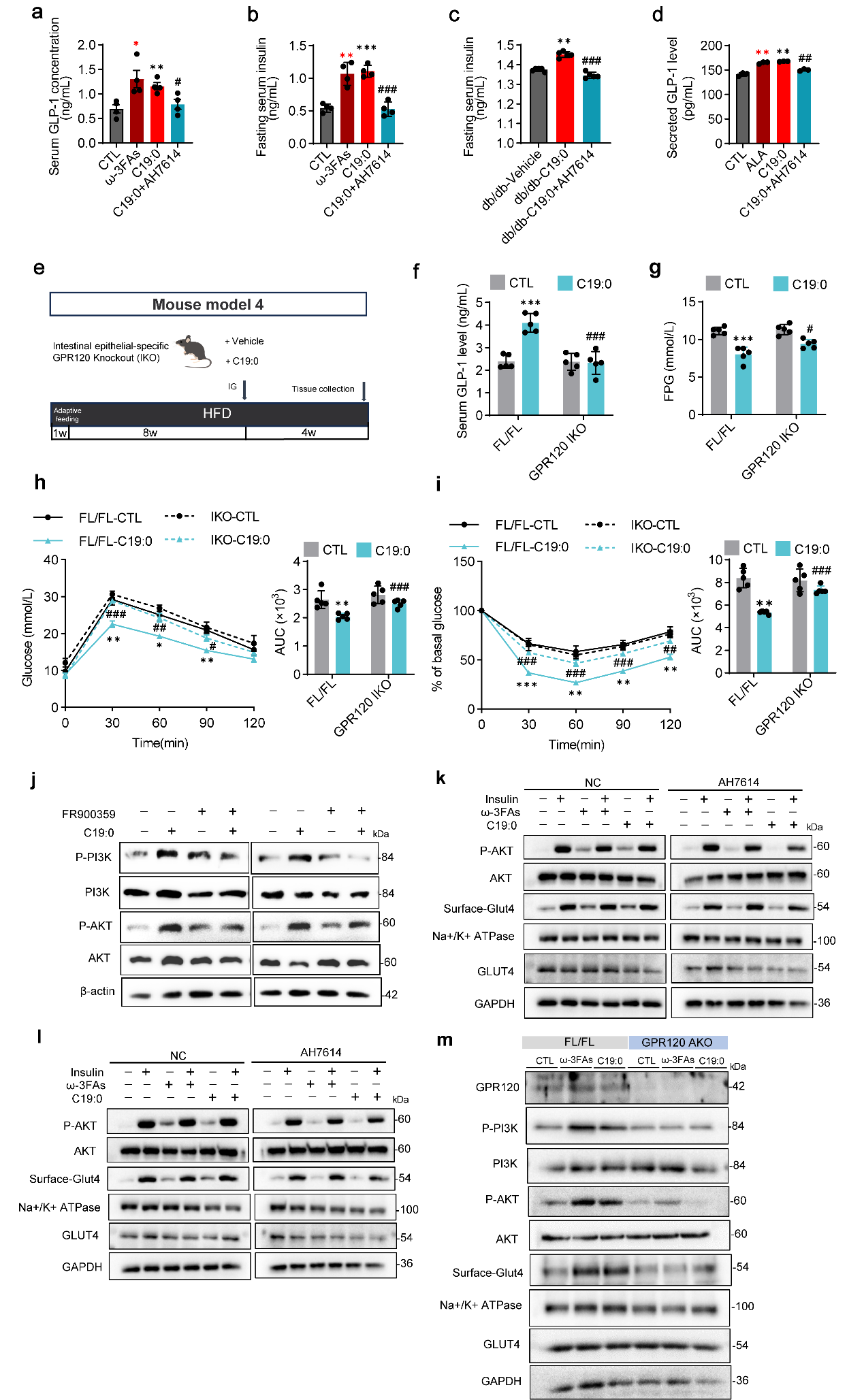


**Figure S4.** C19:0 promotes secretion of GLP-1 and insulin via GPR120-mediated signaling. (a) Serum GLP-1 levels in HFD-fed mice following oral administration of C19:0 (*n* = 4). (b) Fasting serum insulin levels in HFD-fed mice following oral administration of C19:0 (*n* = 4). (c) Fasting serum insulin levels in *db/db* mice following oral administration of C19:0 (*n* = 5). (d) GLP-1 secretion in NCI-H716 cells after 3-h incubation with 100 μM C19:0 (*n* = 3). (e) Mouse model 4, C19:0 oral administration in intestinal epithelial-specific GPR120 Knockout (IKO) mice, mice were treated with vehicle control (CTL) or Nonadecanoic acid (C19:0, 300 mg/kg/day). (f) Serum GLP-1 levels in GPR120 IKO mice (*n* = 5). (g) Fasting plasma glucose levels in GPR120 IKO mice (*n* = 5). (h, i) GTT and its AUC (h), ITT and its AUC (i) (*n* = 5). (j) 3T3-L1 adipocytes endogenously expressing GPR120 were pretreated with FR900359 (1 μM), then stimulated with C19:0. P-PI3K and P-AKT levels were assessed. P-PI3K and P-AKT levels were analyzed. (k, l) phosphorylated AKT (Ser473) and membrane GLUT4 expression in primary adipocytes following treatment with 100 μM DHA or 100 μM C19:0. (m) Protein expression levels of GPR120, P-PI3K, P-AKT, and membrane GLUT4 in WAT of GPR120 AKO mice. Data are shown as mean with SEM. *^,#^*p* < 0.05, **^,##^*p* < 0.01, ***^,###^*p* < 0.001. One-way ANOVA test was performed in (a–d), and a two-way ANOVA test was performed in (f–i).


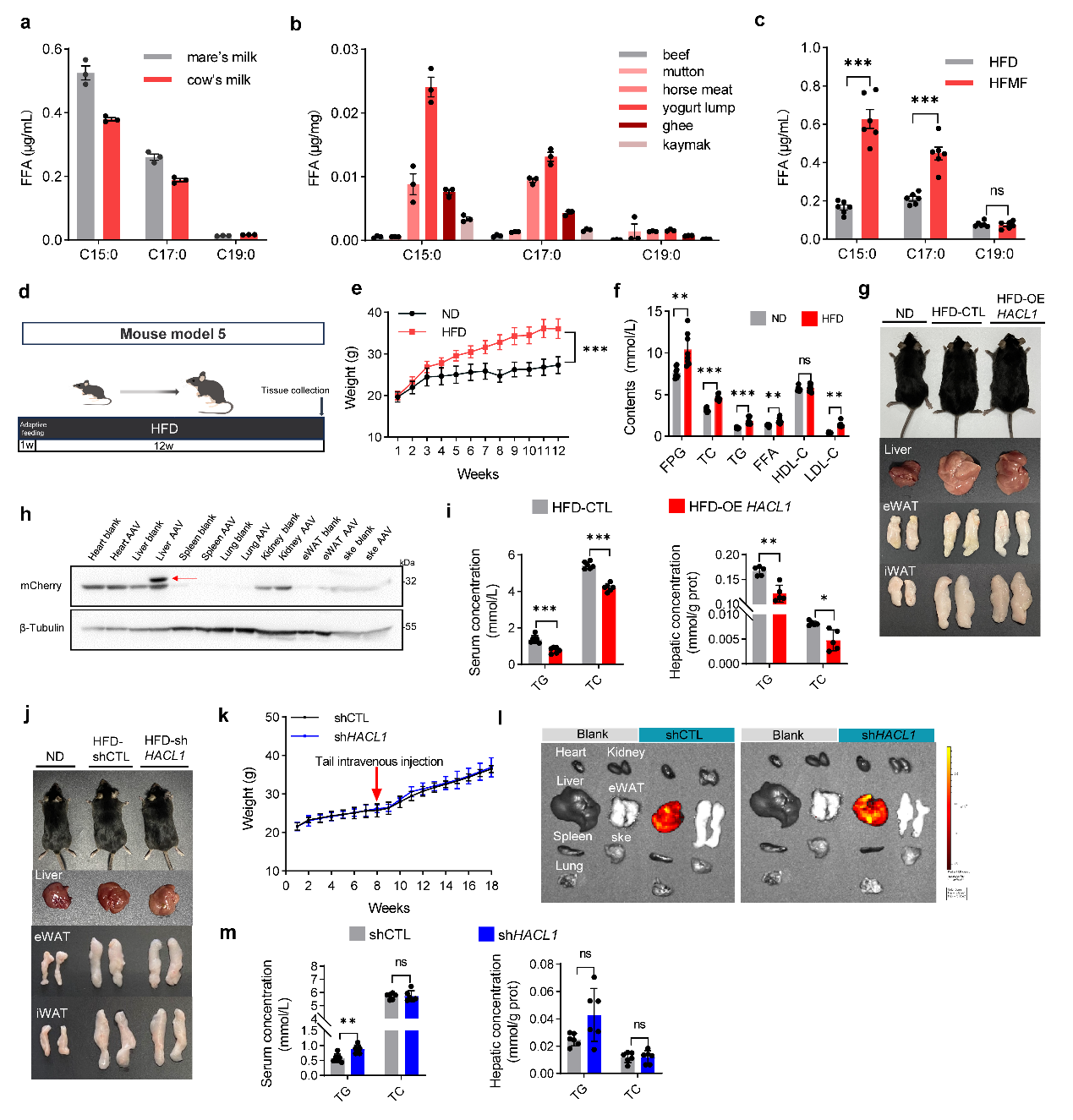


**Figure S5.** Establishment of liver-specific HACL1 overexpression and knockdown models in obese mice. (a) The levels of C15:0, C17:0, and C19:0 in Mare's milk and cow milk (*n* = 3). (b) The levels of C15:0, C17:0, and C19:0 in meat and dairy products commonly consumed by the Kazakh population (*n* = 3). (c) Changes in the levels of C15:0, C17:0, and C19:0 in the serum of mice fed a high-milk-fat diet (high-fat diet supplemented with 14% milk fat, HFMF) (*n* = 6). (d) Mouse model 5: Construction of an obese mouse model. (e) Body weight of HFD-induced obese mice (*n* = 10). (f) Serum levels of fasting plasma glucose (FPG), triglycerides (TG), free fatty acids (FFA), total cholesterol (TC), low-density lipoprotein cholesterol (LDL-C), and high-density lipoprotein cholesterol (HDL-C) in HFD-fed mice (*n* = 8). (g) Representative images of whole-body phenotype, liver, epididymal white adipose tissue (eWAT), and inguinal white adipose tissue (iWAT) from HACL1-overexpressing mice. (h) mCherry expression in various mouse tissues, confirming successful HACL1 overexpression. (i) Serum and hepatic TG and TC levels in HACL1-overexpressing mice. (j) Representative images of whole-body phenotype, liver, eWAT, and iWAT from HACL1-knockdown mice. (k) Body weight progression in HACL1-knockdown mice (*n* = 6). (l) Representative *in vivo* imaging system (IVIS) images of liver and other tissues after 10 weeks of tail vein injection with mCherry-labeled adeno-associated virus (AAV). (m) Serum and liver TG/TC concentrations in HACL1-knockdown mice. Data are shown as mean with SEM. **p* < 0.05, ***p* < 0.01, ****p* < 0.001. Student’s *t*-test was performed in (c, e, f, i, m).


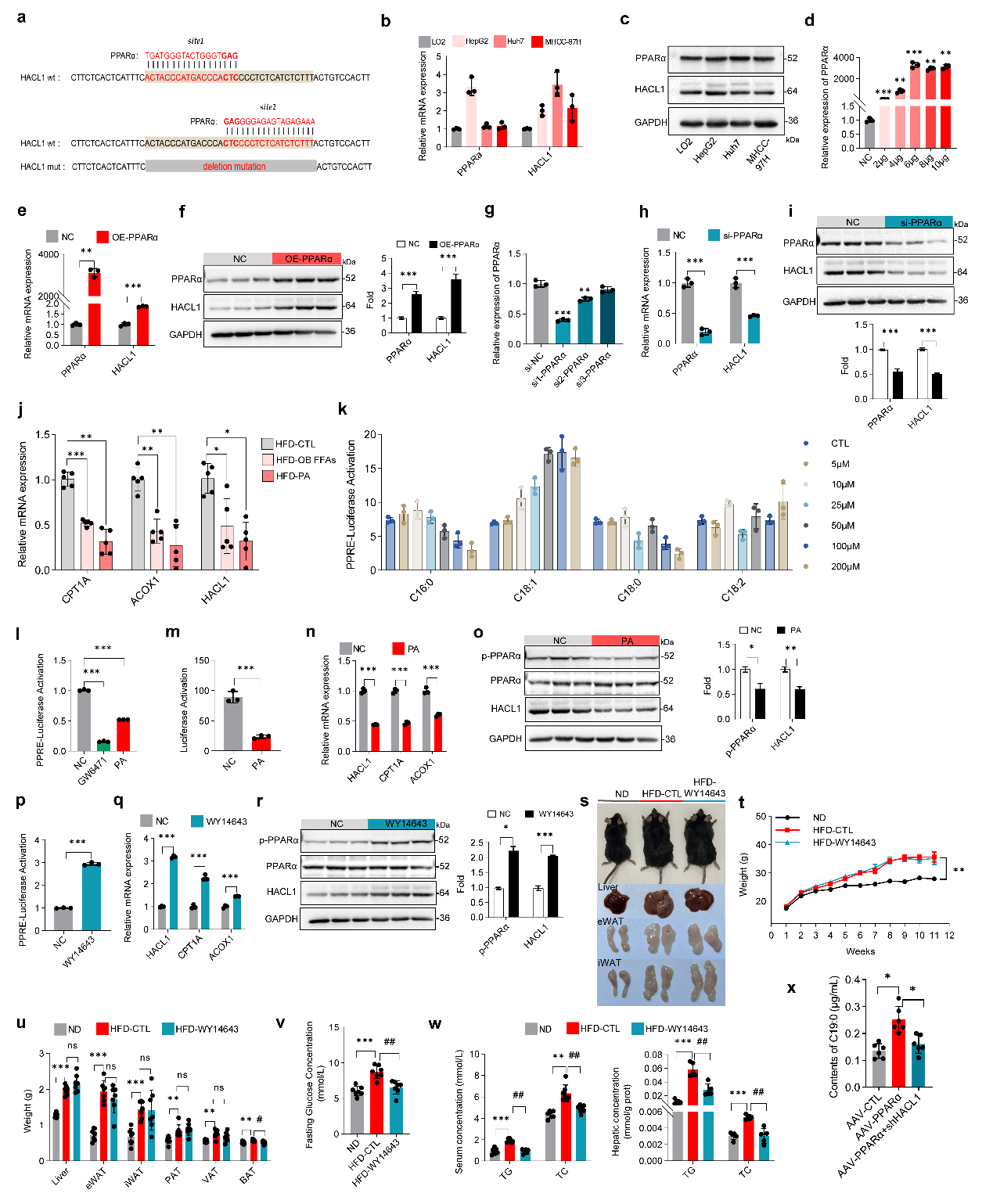


**Figure S6.** PPARα transcriptionally activates HACL1. (a) Schematic representation of two potential PPARα binding sites (red) within the HACL1 promoter region. (b, c) Expression levels of PPARα and HACL1 in liver cell lines LO2, HepG2, Huh7, and MHCC-97H. (d) PPARα mRNA expression in LO2 cells transfected with increasing concentrations of PPARα plasmid. (e, f) mRNA (e) and protein (f) expression levels of PPARα and HACL1 in LO2 cells. (g) PPARα mRNA expression in HepG2 cells transfected with PPARα interference fragments. (h, i) mRNA (h) and protein (i) expression levels of PPARα and HACL1 following transfection with siPPARα in HepG2 cells. (j) Primary hepatocytes isolated from obese mice were exposed to a physiologically relevant fatty acid mixture—comprising 200 μM palmitic acid, 150 μM oleic acid, 30 μM stearic acid, and 50 μM linoleic acid—and the mRNA expression levels of CPT1A, ACOX1, and HACL1 were quantified. (k) PPARα transactivation in HepG2 cells in response to increasing concentrations (5-200 μM) of palmitic acid (C16:0), oleic acid (C18:1), stearic acid (C18:0), and linoleic acid (C18:2). (l) Luciferase reporter assay assessing PA-induced PPARα transcriptional activity using a PPAR response element (PPRE) reporter in HepG2 cells. (m) Effect of PA on luciferase activity of the HACL1 promoter reporter construct. (n, o) mRNA (n) and protein (o) expression levels of HACL1, CPT1A, and ACOX1 (PPARα downstream target genes) and p-PPARα in HepG2 cells treated with 200 μM PA. (p) Luciferase reporter assay assessing WY14643-induced PPARα transcriptional activity using a PPAR response element (PPRE) reporter in HepG2 cells. (q, r) mRNA (q) and protein (r) expression levels of HACL1, CPT1A, and ACOX1, as well as p- PPARα, following 50 μM WY14643 treatment in HepG2 cells. (s) Representative images of mice and their liver, epididymal white adipose tissue (eWAT), inguinal white adipose tissue (iWAT), perirenal fat (PAT), and visceral fat (VAT) following WY14643 treatment. (t) Body weight of mice (*n* = 7). (u) Liver and adipose tissue weights (*n* = 7). (v) Fasting plasma glucose levels (*n* = 7). (w) Triglyceride (TG) and total cholesterol (TC) levels in serum (left, *n* = 7) and liver (right, *n* = 5) of WY14643-treated mice. (x) Rescue experiment demonstrates that PPARα regulates C19:0 synthesis via HACL1 (*n* = 6). Data are shown as mean with SEM. *^,#^*p* < 0.05, **^,##^*p* < 0.01, *** indicates *p* < 0.001, Student’s t-test was performed in (g, h, j, k, m–p, q, r, t) one-way ANOVA test was performed in (f, i, l, v, x), or two-way ANOVA test was performed in (a, u, w).


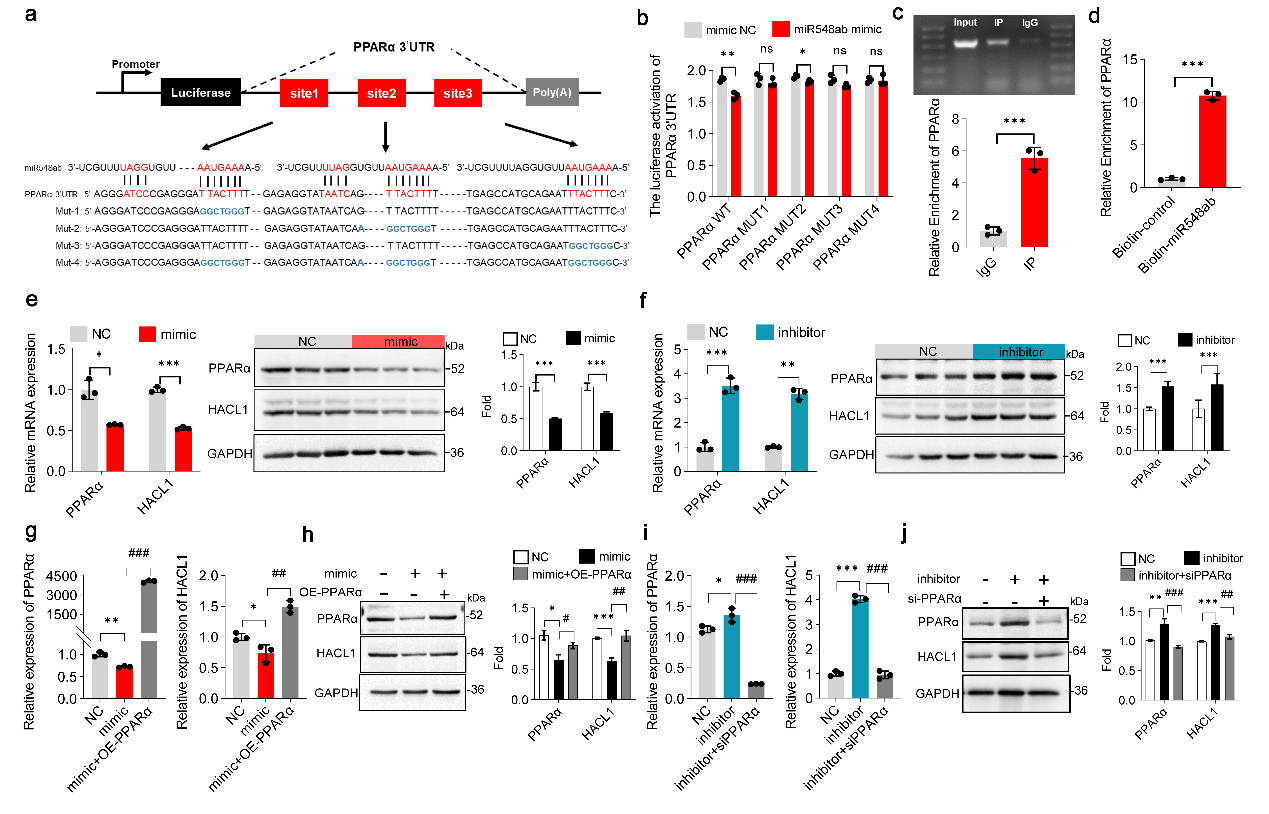


**Figure S7.** miR548ab targets and regulates PPARα expression. (a) Schematic representation of three potential miR548ab binding sites (red) within the PPARα 3′-UTR. Four mutant constructs of the PPARα 3′-UTR were generated by replacing the wild-type binding sequence (AAUGAAA) with a mutant sequence. (b) miR548ab mimic suppresses PPARα 3′ UTR luciferase activity, with binding sites 1 and 3 required for the inhibitory effect. (c) RNA immunoprecipitation (RIP) assay demonstrating the interaction between miR548ab and PPARα. (d) Enrichment of PPARα pulled down by biotin-miR548ab or biotin-control. (e) PPARα and HACL1 mRNA and protein expression levels in HepG2 cells overexpressing miR548ab. (f) PPARα and HACL1 mRNA and protein expression levels in LO2 cells transfected with miR548ab inhibitor. (g, h) Overexpression of miR548ab was accompanied by upregulation of PPARα, mRNA (g), and protein (h) expression of PPARα and HACL1 in HepG2 cells. (i, j) Inhibition of miR548ab was accompanied by downregulation of PPARα, mRNA (i), and protein (j) expression of PPARα and HACL1 in HepG2 cells. Data are shown as mean with SEM. *^,#^*p* < 0.05, **^,##^*p* < 0.01, ***^,###^*p* < 0.001. Student’s *t*-test was performed in (b–f), one-way ANOVA test was performed in (g, i), or two-way ANOVA test was performed in (h, j).


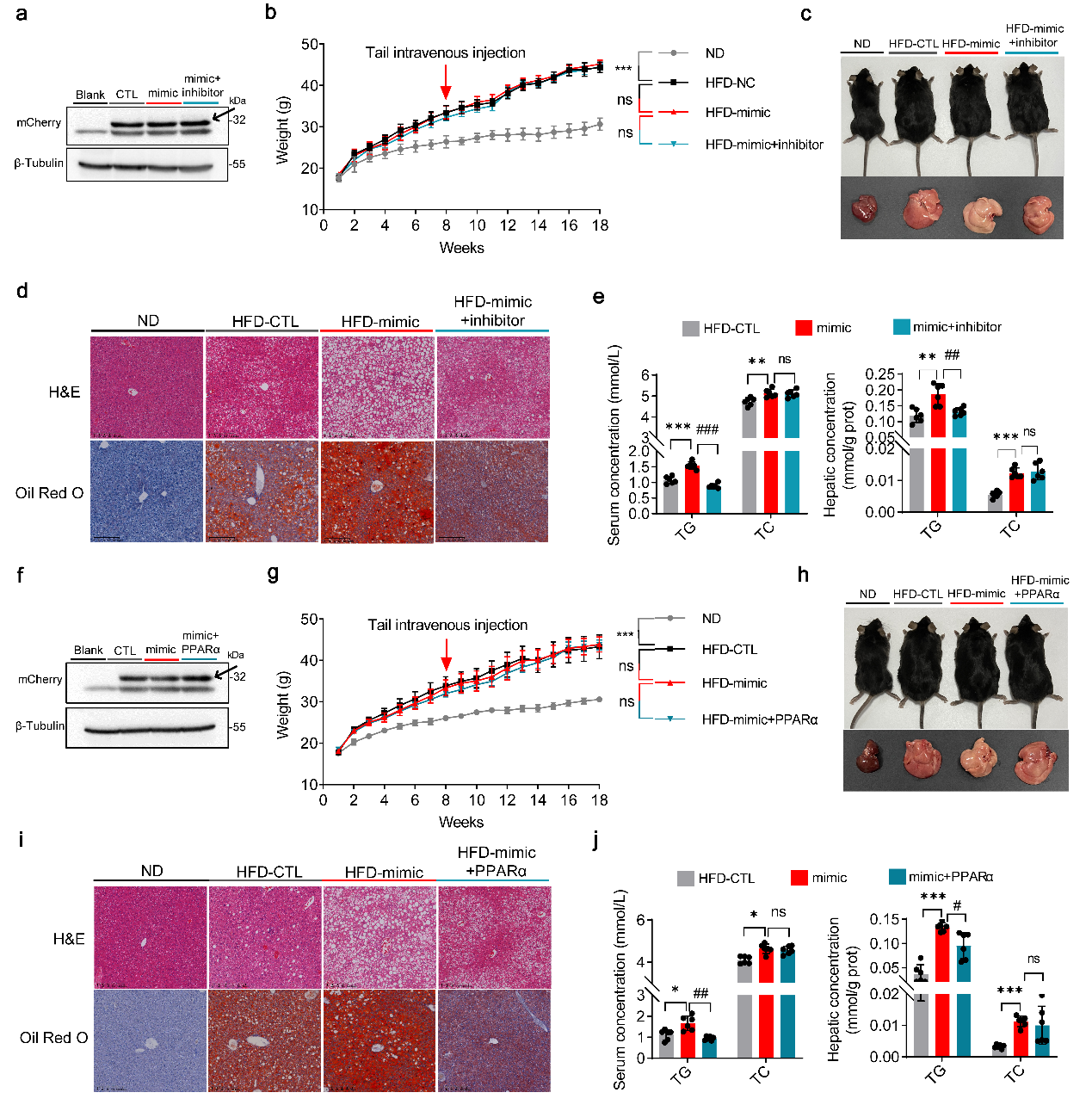


**Figure S8.** miR548ab downregulates hepatic HACL1 expression by inhibiting PPARα. (a, f) mCherry expression in mouse livers. (b, g) Body weight of mice (*n* = 6). (c, h) Representative images of whole mice and liver morphology. (d, i) Representative hematoxylin and eosin (H&E) and Oil Red O staining of liver sections. (e, j) TG and TC levels in serum and liver (*n* = 6). Data are shown as mean with SEM. *^,#^*p* < 0.05, **^,##^*p* < 0.01, ***^,###^*p* < 0.001. Student’s t-test was performed in (b, g), or a two-way ANOVA test was performed in (e, j).


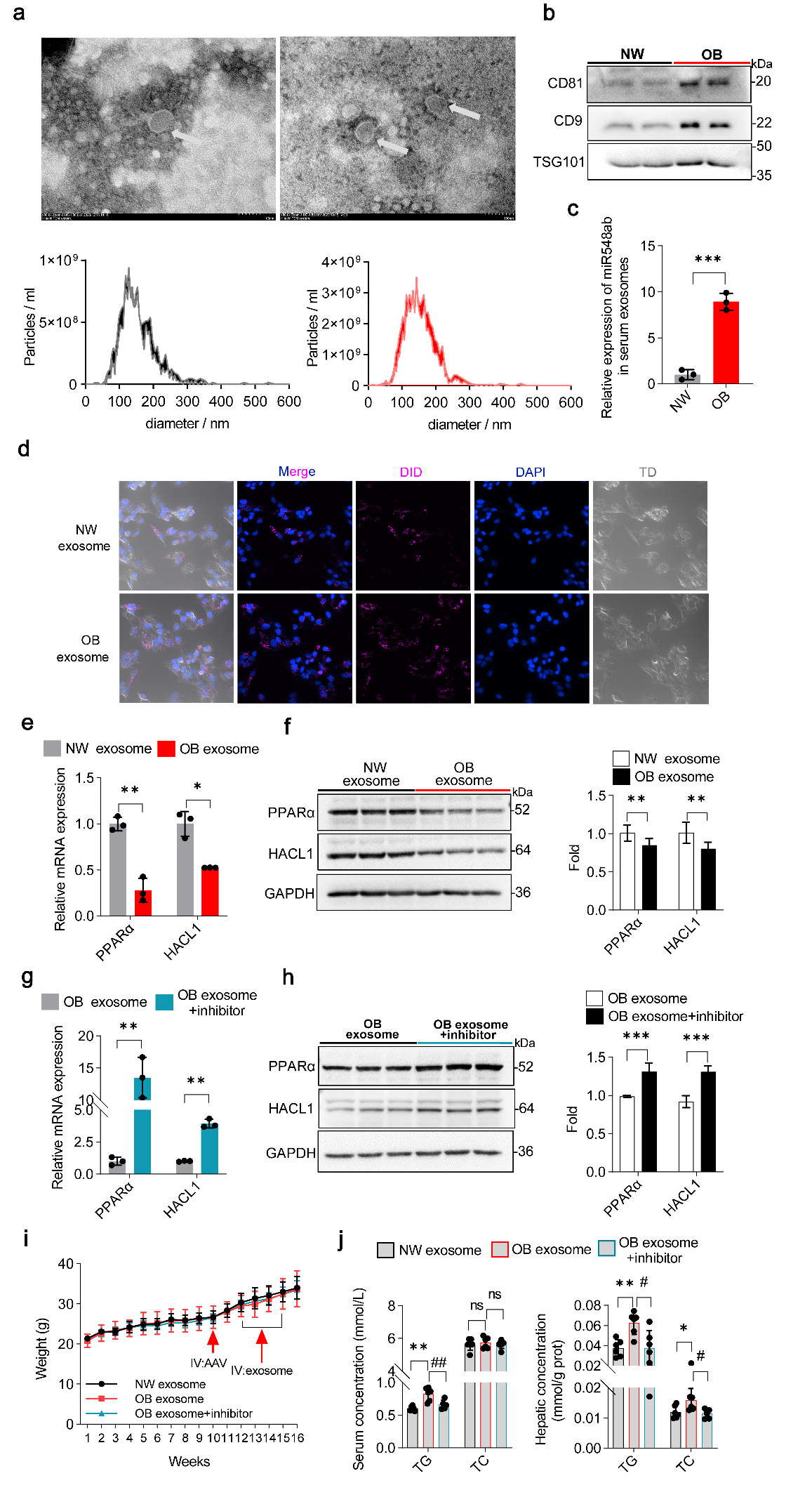


**Figure S9.** miR548ab functions in hepatocytes via exosome-mediated delivery. (a) Representative electron micrographs of serum-derived exosomes (top) and nanoparticle tracking analysis showing the size distribution of serum exosomes from normal-weight (NW) and obese (OB) subjects (bottom). (b) Western blot analysis of exosome marker expression in serum-derived exosomes. (c) miR548ab content in serum exosomes from NW and OB subjects. (d) Representative images of DID-labeled serum exosomes co-incubated with HepG2 cells. (e, f) mRNA (e) and protein (f) expression of PPARα and HACL1 in HepG2 cells following co-incubation with equal amounts of serum-derived exosomes from NW and OB subjects. (g, h) mRNA (g) and protein (h) expression of PPARα and HACL1 in HepG2 cells after co-incubation with OB-derived exosomes and transfection with a miR548ab inhibitor. (i) Body weight of mice (*n* = 6). (j) TG and TC levels in serum and liver (*n* = 6). Data are shown as mean with SEM. *^,#^*p* < 0.05, **^,##^*p* < 0.01, ****p* < 0.001. Student’s *t*-test was performed in (c, e–h), or a two-way ANOVA test was performed in (j).

**Table S1. General characteristics of the subject individuals.**

| **Index** | **Han** | **Uyghur** | **Kazak** |
| --- | --- | --- | --- |
| **Number of Subject** | 3265 | 11403 | 4719 |
| **Age(year)** | 48.97±13.27 | 42.19±12.44^***^ | 43.84±12.62^***###^ |
| **Height(m)** | 1.63±0.08 | 1.60±0.09^***^ | 1.64±0.09^***###^ |
| **Weight(kg)** | 64.02±11.23 | 66.52±12.83^***^ | 67.27±13.66^***###^ |
| **BMI (kg/m^2^)** | 24.03±3.37 | 25.99±4.48^***^ | 25.06±4.41^***###^ |
| **WC (cm)** | 81.07±10.36 | 90.18±12.71^***^ | 85.01±12.24^***###^ |
| **FPG (mmol/L)** | 5.36±1.28 | 5.54±4.39^*^ | 4.59±1.23^***###^ |
| **TG (mmol/L)** | 1.41±1.40 | 2.45±1.61^***^ | 1.02±0.81^***###^ |
| **TC (mmol/L)** | 4.66±0.94 | 4.17±2.17^***^ | 4.89±1.46^***###^ |
| **LDL-C(mmol/L)** | 2.77±0.78 | 2.63±0.88^***^ | 2.64±0.87^***^ |
| **HDL-C(mmol/L)** | 1.44±0.31 | 1.47±0.66^**^ | 1.57±0.41^***###^ |

Abbreviations: BMI, Body mass index; WC, waist circumference; FPG, fasting blood glucose; TG, triglycerides; TC, total cholesterol; LDL, low-density lipoprotein; HDL, high-density lipoprotein. (One-way ANOVA-LSD, t-test, ^*^*P*<0.05, ^**^ *P*<0.01, ^***^ *P*<0.001 vs Han; ^###^ *P*<0.01 vs Uyghur, the difference was statistically significant, data presented as mean ± standard deviation)

**Supplementary Materials**

**KEY RESOURCES TABLE**

| REAGENT or RESOURCE | SOURCE | IDENTIFIER |
| --- | --- | --- |
| Antibodies | | |
| Rabbit polyclonal anti-p-PI3K | Cell Signaling Technology | Cat# 4228  RRID: AB-659940 |
| Rabbit polyclonal anti-PI3K | Abcam | Cat#AB191606  RRID: AB-2891324 |
| Rabbit polyclonal anti-p-AKT | Cell Signaling Technology | Cat# 4060  RRID: AB-2315049 |
| Rabbit polyclonal anti-AKT | Cell Signaling Technology | Cat# 4685  RRID: AB-2225340 |
| Rabbit polyclonal anti-p-ERK1/2 | Proteintech | Cat#28733-1-AP  RRID: AB-2881202 |
| Rabbit polyclonal anti-ERK1/2 | Proteintech | Cat#11257-1-AP  RRID: AB-2139822 |
| Rabbit polyclonal anti-GLUT4 | Proteintech | Cat#66846-1-Ig  RRID: AB-2882186 |
| Rabbit polyclonal anti-p-PPARα | Thermo Fisher | Cat#PA1-820  RRID: AB-325817 |
| Rabbit polyclonal anti-HACL1 | Thermo Fisher | Cat#PA5-28511  RRID: AB-2545987 |
| Mouse monoclonal anti-PPARα | Proteintech | Cat#66826-1-Ig  RRID: AB-2882169 |
| Rabbit polyclonal anti-mCherry | Proteintech | Cat#26765-1-AP  RRID: AB-2876881 |
| Mouse monoclonal anti-GAPDH | ZSGB-BIO | Cat#TA-08  RRID: AB-2747414 |
| Mouse monoclonal anti-β-Tubulin | ZSGB-BIO | Cat#TA-10  RRID: AB-3095964 |
| Anti-rabbit, HRP-linked | ZSGB-BIO | Cat#ZB-2301  RRID: AB-2747412 |
| Anti-mouse, HRP-linked | ZSGB-BIO | Cat#ZB-2305  RRID: AB-2747415 |
| Chemicals, peptides, and recombinant proteins | | |
| C19:0 | Sigma | Cat# 72332  CAS:646-30-0 |
| C16:0 | Sigma | Cat# P0500  CAS:57-10-3 |
| AH7614 | TOCRIS | Cat#5256  CAS:6326-06-3 |
| H89 | MCE | Cat#HY-15979  CAS: 127243-85-0 |
| U73122 | MCE | Cat#HY-13419  CAS: 112648-68-7 |
| PTX | Sigma | Cat# P7208  CAS: 70323-44-3 |
| Pluronic F-127 | Sigma | Cat# P2443  CAS:9003-11-6 |
| WY14643 | Sigma | Cat# C7081  CAS:50892-23-4 |
| recombinant PPARα | CUSABIO | Cat#CSB-BP018421HU |
| TRIzol Reagent | Invitrogen | Cat#15596026CN |
| RIPA buffer | Solarbio | Cat#R0010 |
| Critical commercial assays | | |
| Human HACL1 ELISA | Westang | Cat#FP15519 |
| Mouse HACL1 ELISA | Westang | Cat#F13412 |
| Lipofectamine 2000 | Invitrogen | Cat#11668019 |
| Dual-luciferase Reporter Assay | Promega | Cat#E2920 |
| Mouse Insulin ELISA Kit | Jianglaibio | Cat#JL11459 |
| Revert Aid First Strand cDNA Synthesis Kit | Thermo Fisher | Cat#K1622 |
| SYBR Select Master Mix | Thermo Fisher | Cat#208054 |
| Triglycerides reagent | Jiancheng | Cat#E2920 |
| Experimental models: Cell lines and Organisms/strains | | |
| HEK293T | ATCC | RRID:CVCL_0063 |
| HepG2 | Cell Bank of the Chinese Academy of Sciences | RRID:CVCL_0027 |
| LO2 | Bioharbor Biotechnology | RRID:CVCL_6926  STR profiling confirmed identity; no cross-contamination detected |
| Software and algorithms | | |
| GraphPad Prism | GraphPad Software | http://www.graphpad.com/ |
| Adobe Illustrator | Adobe | https://www.adobe.com/products/illustrator.html |
| SPSS 20.0 | IBM SPSS | https://www.ibm.com/hk-en/products/spss-statistics |
| ImageJ | National Institutes  of Health (NIH) | https://imagej.nih.gov/ij/ |

**Oligonucleotides**

Human PPARα Forward: TCGGCGAGGATAGTTCTGGAAG

Human PPARα Reverse: GACCACAGGATAAGTCACCGAG

Human HACL1 Forward: TATGGTCGTCCAGGTGCTTGCT

Human HACL1 Reverse: TGCACACAGCAGAGGTTTCTGC

Human CPT1A Forward: GATCCTGGACAATACCTCGGAG

Human CPT1A Reverse: CTCCACAGCATCAAGAGACTGC

Human ACOX1 Forward: GGCGCATACATGAAGGAGACCT

Human ACOX1 Reverse: AGGTGAAAGCCTTCAGTCCAGC

Human GAPDH Forward: GTCTCCTCTGACTTCAACAGCG

Human GAPDH Reverse: ACCACCCTGTTGCTGTAGCCAA

Mouse PPARα Forward: ACCACTACGGAGTTCACGCATG

Mouse PPARα Reverse: GAATCTTGCAGCTCCGATCACAC

Mouse HACL1 Forward: CGGACTATGCTTCAGAACTGCC

Mouse HACL1 Reverse: CCAGGACTTCTGTCTTTAGCCAC

Mouse CPT1A Forward: GGCATAAACGCAGAGCATTCCTG

Mouse CPT1A Reverse: CAGTGTCCATCCTCTGAGTAGC

Mouse ACOX1 Forward: GCCATTCGATACAGTGCTGTGAG

Mouse ACOX1 Reverse: CCGAGAAAGTGGAAGGCATAGG

Mouse β-actin Forward: CATTGCTGACAGGATGCAGAAGG

Mouse β-actin Reverse:TGCTGGAAGGTGGACAGTGAGG
